# Heat stress in dairy buffalo: biometeorological, molecular, and adaptive strategies for climate change resilience in subtropical regions

**DOI:** 10.1007/s11259-025-11009-y

**Published:** 2026-01-16

**Authors:** Eman M. Ismail, Aly M. Aly, Heba S. Farag, Shaimaa Kamel, Karima M. Fahim

**Affiliations:** 1 Department of Veterinary Hygiene and Management, Faculty of Veterinary Medicine, Cairo University, Giza, 12211 Egypt; 2 Department of Natural Resources, Faculty of African Postgraduate Studies, CU, Giza, 12613 Egypt; 3 Department of Internal Medicine and Infectious Diseases, Faculty of Veterinary Medicine, CU , Giza, 12211 Egypt; 4 Department of Biochemistry and Molecular Biology, Faculty of Veterinary Medicine, CU, Giza, 12211 Egypt; 5 Department of Food Hygiene and Control, Faculty of Veterinary Medicine, CU, Giza, 12211 Egypt

**Keywords:** *Bubalus bubalis*, Temperature‒humidity index (THI), Physiological indices, Milk yield and quality, Energy homeostasis genes, Adaptive management strategies

## Abstract

Buffalo milk production in Egypt has steadily declined since 2014, mainly due to climate-driven heat stress (HS) and rising temperature–humidity index (THI). This quasi-field study randomly evaluated twelve lactating buffalo during peak summer, introducing a biometeorological approach to define and predict HS impacts precisely. Heat stress in buffalo was classified according to THI ranges as follows: non-HS zone (NHSZ, 56.7–73.2), moderate HS zone (MHSZ, 73.2–75.4), severe HS zone (SHSZ, 75.4–80.3), and critical HS zone (CHSZ, ≥ 80.3). Two models were compared: Model I (natural, group A) and Model II (adaptive, group B), which received targeted environmental and management interventions. Continuous monitoring of THI alongside daily milk yield (DMY), physiological responses, oxidative stress biomarkers, and the expression of key energy homeostasis genes was assessed in both groups. Adaptive interventions effectively reduced THI exposure, shifted animals from critical HS to non-HS zones, improved physiological parameters, increased milk yield by 53%, lowered oxidative stress, and enhanced milk quality (*p <* 0.05). The study presents the first transcriptional analysis of stress-responsive energy-regulating genes in buffalo, revealing higher *AMPK*, *HRH1*, and *mTOR* expression in HS-Model I buffalo, which reflects the metabolic strain associated with unmanaged thermal stress. Regression analysis showed that for every one-unit increase in THI above 69, milk yield decreased by 0.17–0.23 kg/day. These findings underscore the value of integrated biostatistical modeling and targeted adaptation strategies for sustaining buffalo productivity under the pressures of subtropical climates. Adaptive housing, nutritional support, and management interventions effectively mitigate the impacts of HS. At the molecular level, evidence of oxidative stress and altered energy regulation highlights the physiological toll of thermal load, emphasizing the need for holistic approaches to protect productivity and herd resilience in heat-stressed regions.

**Supplementary Information:**

The online version contains supplementary material available at 10.1007/s11259-025-11009-y.

## Introduction

Global warming poses a threat to buffalo dairy production, particularly in subtropical regions where summer temperatures exceed the thermal comfort zone of buffalo (Singh et al. 2015; Rabie 2020; Zhang and Jeong 2022). Domestic water buffalo (Bubalus bubalis) play a crucial role in the dairy sector of many developing countries, including Egypt, and rank among the top ten buffalo milk producers worldwide (Marai and Habeeb 2010). In Egypt, buffalo constitute more than 70% of the country’s annual milk supply, which is attributed to their high milk yield and adaptability (Arefaine and Kashwa 2015; Minervino et al. 2020; Rabie 2020). However, climate change has increasingly threatened buffalo productivity in recent years. Since 2014, raw buffalo milk yields in Egypt have declined steadily from 2.9 million tons in 2014 to 1.27 million tons in 2022 (FAO FAOSTAT 2022), attributed mainly to rising temperatures, shifting climate dynamics, resource limitations, and inadequate adaptive management (Omran 2024). Understanding these mechanisms and developing effective mitigation strategies is critical to ensuring sector sustainability and national food security. Addressing these challenges and adaptation strategies is crucial for ensuring sector sustainability, safeguarding food security (Baumgard and Rhoads 2013; Das et al. 2016; Rabie 2020; Omran and Fooda 2023; Omran 2024; Slayi and Jaja 2025).

Heat stress occurs when environmental factors, such as temperature and humidity, exceed an animal’s comfort zone (Smith et al. 2019). Consequently, HS disrupts physiology and behavior, reduces productivity, milk yield, and reproductive performance, and increases disease susceptibility (Collier et al. 2017; Polsky and von Keyserlingk 2017). Reducing HS risk in dairy buffalo begins with the development of robust biostatistical models that predict thermal load using reliable indicators such as the temperature–humidity index (THI). Several models were adopted for the calculation of THI and have long been employed to forecast milk-yield variations and physiological responses under thermal stress (National Research Council (NRC) 1971; Bouraoui et al. 2002; Mader et al. 2006; Dash et al. 2015; Berman et al. 2016; Dash et al. 2016; Purohit et al. 2020; Behera et al. 2023). Beyond its predictive role, THI serves as a practical biometeorological tool for assessing the impact of HS on livestock health, milk production, and composition (Liu et al. 2017; Zhang and Jeong 2022). The respiration rate (RR) regulates body temperature and increases under HS (Omran et al. 2020). HS also induces oxidative stress, particularly in high-producing animals such as buffalo, by increasing reactive oxygen species (ROS) beyond the capacity of natural antioxidants (Waiz et al. 2016; Hady et al. 2018; Sharma et al. 2023). Oxidative stress can be assessed through biomarkers such as malondialdehyde (MDA), glutathione (GSH), and catalase, which indicate the level of stress and the animal’s protective response (Hady et al. 2018; Nandi et al. 2019). Lowering THI values through adaptive practices is therefore essential, particularly when combined with accurate biological-response assessment that explains animal–environment interactions and guides effective mitigation strategies (Omran and Fooda 2023).

Slayi and Jaja (2025) identified three key approaches to mitigate HS in dairy livestock: environmental modification (shading, cooling), improved nutrition, and genetic selection for heat-tolerant breeds. Environmental and nutritional adjustments are the most effective for buffalo. Omran (2024) highlighted the need for adaptation studies for mitigating the impacts of HS on Egyptian buffalo. Hence, our study aimed to present an integrated biometeorological, biochemical, and molecular approach to assess HS resilience in Egyptian buffalo (B. bubalis), a population for which such a comprehensive evaluation has not been previously reported. The approach involved daily monitoring of meteorological indices, milk production, and physiological responses with biochemical, molecular, and milk quality assessment. At the molecular level, the study focused on the expression of key energy homeostasis–related genes (*AMPK*,* HRH1*, and *mTOR*) in milk somatic cells to elucidate buffaloes’ adaptive molecular responses to thermal load. Two HS models were designed to modulate THI and mitigate severe and critical HS zones, offering adaptive environmental, nutritional, and management interventions that aim to enhance buffalo productivity and resilience under subtropical climatic conditions.

## Materials and methods

### Study design and duration

This observational field study aimed to evaluate short-term adaptive strategies for mitigating heat stress in lactating buffaloes. The trial was conducted over 47 consecutive days, from 1 st September to 17th October 2023, during peak summer in Mit Helfa Province, Qalyub, Egypt (N “30°09’59.0976”, E “31°14’34.1196”). Twelve primiparous, late-lactation buffalo (4.8 ± 0.6 years of age) with an average body weight of 552 ± 7.6 kg (Group A) and 553 ± 5.09 kg (Group B) were randomly selected from a large herd and allocated equally into two groups. The sample size was determined using Biomath (2023) software, ensuring sufficient statistical power. All animal handling and management procedures adhered to good veterinary practices (GVPs) to ensure safe and humane milking. Late-lactation buffaloes were selected to minimize confounding effects related to high metabolic demand and hormonal variation typical of early- and mid-lactation stages.

## Comparative housing models and microclimatic management

Twelve buffaloes were allocated into two groups to evaluate the effectiveness of adaptive management interventions against natural environmental exposure in various heat stress (HS) zones.

### Environmental modelling and animal groups

#### Group A (Natural exposure- model I)

Six buffalo were maintained under conventional farming conditions, representing typical buffalo dairy operations. Animals were housed in an open barn with unrestricted access to an unshaded outdoor yard, standing on earthen flooring without mechanical ventilation or cooling systems. The management protocol followed traditional grazing patterns from sunrise to sunset. Daily basal diets were formulated on a total dry matter basis. They consisted of corn fodder (50%), barley straw (16.8%), flaxseed hulls (16.8%), and a mixed concentrate (16.5%). The mixed concentrate consisted of ground yellow corn, wheat bran, soybean meal, and cottonseed cake, supplemented with a commercial mineral–vitamin premix. The overall ration provided 8.4% crude protein (CP), 10.47% total digestible nutrients (TDN), 45% neutral detergent fiber (NDF), and 33% acid detergent fiber (ADF), with mineral content including 0.45% calcium (Ca), 0.4% phosphorus (P), 1.5% potassium (K), and 50 ppm manganese (Mn), following the recommendations of Moran (2005). The feeding practices and nutrient formulations were designed to meet the maintenance and lactation requirements of late-lactation buffalo under field conditions.

#### Group B (Adaptive management- model II)

Six buffalo were housed in a climate-modified facility (8 m × 9 m × 3 m) designed according to the Guidelines for Buffalo Farming (Arab Organization for Agricultural Development 2022). Animals receive controlled outdoor access, limited to two hours daily at sunset. The housing system incorporated species-specific welfare provisions, including insulation, enhanced ventilation, and active cooling interventions, to optimize microclimatic conditions and reduce thermal loads.

### Environmental interventions and nutritional mitigation protocols

Natural Exposure (Group A): Buffalo experienced direct solar radiation exposure from morning through noon during field grazing, with continued heat exposure in the open barn facility during evening periods.

Adaptive strategies (Group B), cooling Protocol: the modified housing system featured comprehensive thermal management including: shaded insulated structure with ceiling curtains, cement roofing covered with dry plant material for additional insulation, enhanced cross-ventilation through two mesh-covered windows, dual large-capacity ventilation fans for continuous air circulation, misting system operation for 4–6 h daily pre-milking, and twice-daily cooling treatments using wet towels for 30 min before milking sessions. The use of wet towels was selected as a practical evaporative cooling technique commonly adopted by smallholder farmers in tropical regions, aimed at reducing pre-milking body temperature without requiring advanced infrastructure.

In addition to the cooling protocol, Group B received targeted nutritional supplementation designed to support metabolic stability and resilience under heat stress conditions. Enhanced feeding protocols included standard rations supplemented with multivitamins (1 g/L), vitamin C (1 g/L), and a rumen booster (100 g/total ration, administered twice daily). The supplementation formulation incorporated essential vitamins (A, D₃, E, K₃, and B-complex), minerals (Ca, Mg, Mn, Zn, K, Na), amino acids (lysine and methionine), and probiotics (*Lactobacillus*, *Bifidobacterium*, *Streptococcus*, and *Enterococcus*) (Table 1) (Das et al. 2016; Nzeyimana et al. 2023; Kausar and Imran 2024). These supplements were provided to enhance nutrient utilization and maintain physiological performance during exposure to elevated temperature and humidity conditions. Daily facility sanitation protocols ensured continuous access to fresh, clean water sources.

**Table 1:** Targeted feed and water supplementation for the alleviation of HS in the adaptive model II

Additive ingredients	Supplementation concentration
Multivitamins	One g/Liter of drinking water
Vitamin C (20%)	One g/L
Rumen boosters + Levabon^®^ Rumen	100 gm/total ration/twice daily
Vitamin C	1000 mg/g
Vitamin A (retinyl acetate)	15,000,000 Iu
Vitamin D3 (cholecalciferol)	4,400,000 Iu
Vitamin E (alpha-tocopheryl acetate)	1350 mg/g
Vitamin K3 (MSB)	4350 mg/g
Vitamin B2	4350 mg/g
Vitamin B6	2350 mg/g
Vitamin B12	10,000 mcg
Nicotinic acid	16,700 mg/g
Multimix^®^ (minerals)	One g/kg
limestone (calcium carbonate)	400 mg/kg
Calcium pantothenate	5350 mg/g
Magnesium sulfate calcined	2376 mg/g
Manganese sulfate monohydrate	3840 mg/g
Zinc sulfate monohydrate	7999 mg/g
Potassium chloride	45,627 mg/g
Sodium sulfate	68,625 mg/g
Sodium chloride	19,669 mg/g
Lysine monohydrochloride	15,000 mg/g
DL-Methionine	10,000 mg/g
Lactose (monohydrate)	up to 1 kg
Lactobacillus species	6.00 × 10^11^ Colony Forming Unit (CFU)
*Lactobacillus plantarum*	3.78 × 10^9^ CFU
*Lactobacillus delbrueckii* subsp. Bulgaricus	6.18 × 10^9^ CFU
*Lactobacillus acidophilus*	6.18 × 10^9^ CFU
*Lactobacillus rhamnosus*	6.18 × 10^9^ CFU
*Bifidobacterium bifidum*	6 × 10^9^ CFU
*Streptococcus salivarius* subsp. Thermophilus	1.23 × 10^10^ CFU
*Enterococcus faecium*	1.94 × 10^10^ CFU

### Environmental monitoring and heat stress assessment

#### Thermal-humidity index (THI) calculation

Defining HS zones was done after calculating THI daily. A dual approach of calibrated indoor sensors and outdoor meteorological station data was adopted for generating a continuous THI profile for both groups, ensuring high spatial accuracy and continuous representation of climatic conditions. The indoor barn temperature (Tb, °C) and relative humidity (RH%) were measured using a digital temperature and humidity data logger (ISO 1998; ASHRAE 2019). Instruments were positioned at the level of the animals (approximately 1.5 m above ground) to capture the microclimate experienced by the buffalo. They were placed away from direct sunlight, water sources, and ventilation outlets in shaded areas where the animals were housed, to avoid bias in readings. Automated systems were calibrated and configured to record data at 30-minute intervals, enabling continuous monitoring of Tb (°C) and RH (%) data, which were used for calculation of THI on an average daily basis (Dash et al. 2016; Behera et al. 2023).

$$\:THI=0.8\times\:{T}_{b}+RH\mathrm{\%}\:\left({T}_{b}-14.4\right)+46.4$$

THI: temperature humidity index; Tb: indoor barn temperature (°C); RH%: relative humidity percent.

For outdoor THI calculation, deploying sensors with sufficient spatial accuracy in broad or inaccessible areas is often challenging. In such cases, meteorological data were obtained from the nearest local ground meteorological station, Banha Station (IBANHA3), approximately 2.5 km from the study site (World Meteorological Organisation (WMO), 2023). Banha Station (IBANHA3) provides hourly meteorological data from which the ambient air temperature (Ta, °C) and dew point temperature (Dp, °C) were obtained and averaged daily to calculate RH% and THI using the standard equations of Mader et al. (2006), Dash et al. (2016), and Behera et al. (2023).

$$\begin{array}{l}RH\%\\=100\times\left\{exp\left\lfloor17.625\times DP/\left(243.04+Dp\right)\right\rfloor/exp\left\lfloor17.625\times T_a/\left(143.04+T_a\right)\right\rfloor\right\}\end{array}$$

$$\:THI=0.8\times\:{T}_{a}+RH\%\:\left(T-14.4\right)+46.4$$

RH%: relative humidity percent; Dp: dew point; THI: temperature humidity index; Ta: ambient temperature (°C).

These models integrate ambient temperature and RH% to quantify the thermal load and classify HS zones with high predictive accuracy. The data presented in the results represent the daily mean ± SE derived from these continuous recordings throughout the 47-day experimental period (1st September–17th October 2023). Complete raw datasets and THI profiles are available in Online Resource (1).

#### Heat stress modeling and zone classification

HS modeling and classification in natural and adaptive groups were based on calculated THI values. HS in buffalo can be categorized into four zones: the non-heat stress zone (NHSZ) (THI 56.71–73.21), the moderate HS zone (MHSZ) (73.22–75.39), the severe HS zone (SHSZ) (75.40–80.27), and the critical HS zone (CHSZ) (≥ 80.27). Buffalo in Group A experienced natural CHSZ, SHSZ, and MHSZ conditions based on THI thresholds (Dash et al. 2014; Dash et al. 2015; Dash et al. 2016; Behera et al. 2023). Group B (adaptive, Model II) resided in an adaptive barn with lower temperatures and relative humidity Tb and RH), thus mainly remaining within the NHSZ (Online resource 1).

### Physiological assessment and production performance

Daily measurements of buffalo rectal temperature (RT, °C) and respiratory rate (RR, breaths per minute) were recorded twice daily—30 min before routine morning (6:00 a.m.) and evening (6:00 p.m.) milking sessions—to minimize handling or milking stress while capturing diurnal thermal variation. Milk yield (kg) was recorded at both milking times, and daily milk yield (DMY) was calculated as the sum of the two measurements. The values presented in the results tables represent daily mean ± standard error (SE) derived from these observations for each buffalo across the 47-day experimental period (1st September–17th October 2023). Detailed daily records are available in the Supplementary Files (Online Resources 2, 3, and 4).

#### Collection of milk samples

Individual composite milk samples were aseptically collected from lactating buffalo **(**Fahim et al. 2019). The teat ends were washed, dried, and swabbed with 70% ethyl alcohol. The first 2–3 streams of milk were discarded, and then the milk samples were collected in sterile vials. The samples were collected and transferred to an insulated icebox, then sent to the laboratory for immediate analysis. The physical characteristics of the milk samples were determined using an electrical conductivity (EC) standard Professional Conductivity-Temp portable meter (AD310, ADWA Instruments Kft., Hungary), with an accuracy of ± 1% (EC) and ± 0.5 °C. Additionally, the pH of the milk was measured using a calibrated professional pH-ORP-Temp portable meter (AD111, ADWA Instruments Kft., Hungary), with an accuracy of ± 0.02 pH.

#### Milk quality assessment

The milk somatic cell count (SCC) (cells/ml), fat %, solid nonfat (SNF %), total solid (TS %), protein %, salt %, lactose %, density, and freezing point (°C) were determined via a Lactoscan Compo Somatic Cell Counter And Ultrasonic Milk Analyzer (MCCP-CB-025597, Milkotronic LTD, Bulgaria). The total bacterial count (TBC) (colony-forming unit/ml) (CFU/ml) was determined by spreading 0.1 ml from the original homogenate and each tenth-fold decimal dilution separately over the surface of double plates of standard plate count agar and then incubating at 30 °C for 48 h (ISO 2013).

### Biochemical and molecular assessment of oxidative- and stress-related genes

The following oxidative stress biomarkers were detected in the collected milk samples: Malondialdehyde (MDA), a marker of lipid peroxidation, was determined according to the method described by Ohkawa et al. (1979). MDA reacts with the thiobarbituric acid reagent in an acidic medium, producing a red-colored pigment that is measured spectrophotometrically at 532 nm against a reagent blank. Reduced glutathione (GSH) was quantified as described by Ellman (1959). The method is based on the reduction of 5,5′-dithiobis-2-nitrobenzoic acid (DTNB) by GSH, forming a yellow-colored chromogen whose absorbance is read at 412 nm. Catalase activity, an indicator of antioxidant defense, was assessed using the method described by Aebi (1984). The enzyme decomposes hydrogen peroxide into water and oxygen, and its activity was measured kinetically by monitoring the decrease in absorbance at 240 nm during incubation with hydrogen peroxide.

Meanwhile, the expression levels of the *AMPK*,* HRH1*, and *mTOR* genes were analyzed as a novel molecular approach to assess stress adaptation in buffalo. Milk samples were first centrifuged at 12,000 rpm for 15 min at 4 °C to separate the somatic cell pellet, which was used for RNA extraction using the ABT Total RNA Mini Extraction Kit (Applied Biotechnology Co. Ltd., Egypt) according to the manufacturer’s protocol. RNA concentration and purity were determined spectrophotometrically at 260 and 280 nm, respectively (Kamel et al. 2018; Mohamed et al. 2024

). Complementary DNA (cDNA) was synthesized using the ABT H-minus cDNA Synthesis Kit (Applied Biotechnology Co. Ltd., Egypt). Quantitative real-time PCR (qRT-PCR) was performed using ABT 2× SYBR Green Master Mix (Applied Biotechnology Co. Ltd., Egypt) to determine the relative expression of *AMPK*,* HRH1*, and *mTOR*, with *β-actin* as the reference gene. The qRT-PCR primers were designed using the primer designing tool, NCBI (https://www.ncbi.nlm.nih.gov/tools/primer-blast/index.cgi?LINK_LOC=BlastHome). The expression level of each gene was calculated via the equations ΔCT, ΔΔCT, and 2 − ΔΔCT (Livak and Schmittgen 2001; Abdel-Gawad et al. 2023). The primer sets for each gene, along with their accession numbers, are listed in Table 2. Each SYBR Green assay was performed in triplicate (Hassanen et al. 2024).

**Table 2:** Primer sets used for qRT‒PCR for the detection of the *AMPK*,* HRH1*, and *mTOR* genes

Gene symbol	Gene description	Accession number	Primer Sequence
*β-actin*	Beta-actin	*NM_001290932.1*	F: 5’ -GGAATCCTGCGGTATTCACGA-3’ R: 5’- GCCTAGAAGCATTTGCGGTG-3’
*AMPK*	protein kinase AMP-activated catalytic subunit alpha (PRKAA1)	*XM_006053837.4*	F: 5’ -TTCTCGACGATCACCACTTGAC- 3’ R: 5’ -TCTTCCTCCGAACACGCAAATA- 3’
*HRH1*	histamine receptor H1	*XM_044933450.2*	F: 5’- ATCAACCAGAGCCAGAACCG − 3’ R: 5’ - ATGTGCAAGCCAGACACGTA − 3’
*mTOR*	Mechanistic target of rapamycin kinase	*XM_025285881.2*	F: 5’- CAGGGACTTGATGGAGGAGAAAT- 3’ R: 5’- ACCTCACAGCCACAGAAAGTAG- 3’

### Statistical and data analysis

Data are expressed as mean ± standard error (SEM). Statistical analyses were conducted using PASW Statistics version 24.0 (SPSS Inc., Armonk, NY, USA). Continuous variables were compared using one-way ANOVA followed by the least significant difference (LSD) post-hoc test for pairwise group comparisons. In contrast, categorical variables were analyzed using the chi-square test. Associations between the THI, physiological parameters, and milk yield were examined using Pearson correlation and linear regression analyses. Statistical significance was set at *p* < 0.05; all other differences were considered non-significant.

## Results

### Environmental, meteorological assessment, and their impact on milk production in buffalo

The comparative assessment of microclimatic conditions in Table 3 reveals a significant difference between natural outdoor and adaptive indoor environments (p < 0.05). Outdoors, the mean temperature (Ta) was 32.23 ± 0.44 °C with a RH% of 40.46 ± 0.90%, yielding a THI of 79.30 ± 0.47. By contrast, indoor adaptive conditions were cooler (26.43 ± 0.38 °C) and less humid (37.90 ± 1.07%), resulting in a lower THI of 72.02 ± 0.42. Statistical analysis confirmed these differences were highly significant (p < 0.05), indicating that adaptive housing and management strategies effectively mitigated buffalo exposure to heat stress compared to natural outdoor conditions. Additional data are provided in the online resource (1).

**Table 3:** Meteorological assessment and THI mean values in natural and adaptive Buffalo groups during the study period (1st September – 17th October 2023)”

Parameter	Natural (HS-model I)	Adaptive (HS-model II)
Temperature (Ta&Tb) (°C)	32.23 ± 0.44^**a**^	26.43 ± 0.38^**b**^
Relative humidity % (RH%)	40.46 ± 0.90^**a**^	37.90 ± 1.07^**b**^
Dew Point (DP) (°C)	16.92 ± 0.35	—
Temperature-Humidity Index (THI)	79.30 ± 0.47^**a**^	72.02 ± 0.42^**b**^

Temperature–humidity index (THI) values correspond to daily averages generated from automated 30−minute interval recordings throughout the 47−day experimental period (1st September– 17th October 2023); Data are presented as mean ± SE. Superscript letters (a, b) indicate significant differences between groups (***p*** < 0.05); Ta, outdoor ambient temperature; Tb, indoor barn temperature. Meteorological data were obtained from the continuous profile in the Online resource (1)

### Heat stress modelling and adaptation strategies

**Fig. 1:**
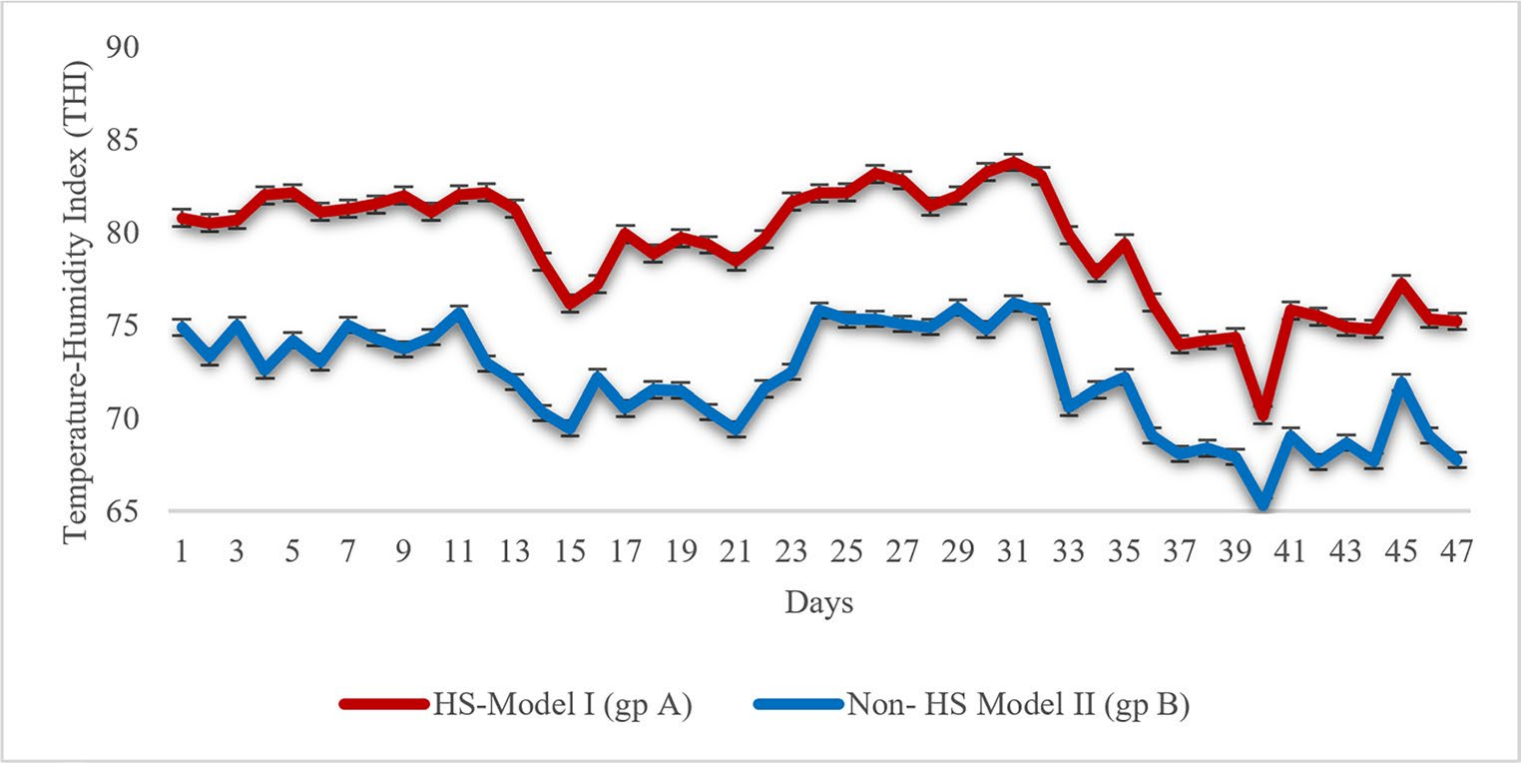
Heat stress modeling based on temperature‒humidity index (THI) profile values over the study period in natural (HS Model I) and adaptive (HS Model II) buffalo groups

Heat stress (HS) of buffaloes was classified with THI threshold values into four zones: non-heat stress zone (NHSZ; THI 56.71–73.21), moderate heat stress zone (MHSZ; THI 73.22–75.39), severe heat stress zone (SHSZ; THI 75.40–80.27), and critical heat stress zone (CHSZ; THI ≥ 80.27). Figure 1 provides the daily THI profile of natural (HS Model I, Group A) and adaptive (HS Model II, Group B) buffalo housing models during the entire study period. Over the 47-day study duration **(**Table 4**)**, buffaloes living outdoors under natural conditions suffered long-term exposure to high THI values (mean 79.30 ± 0.47), with most of the days remaining inside the moderate to critical HS zones, with 29 days inside the CHSZ (80.80–83.80), which was indicative of repeated and long-term exposure to environmental heat stress. Compared with that, the indoor adaptive design significantly lowered THI (mean 72.02 ± 0.42), shifting most of the days into the MHSZ to NHSZ of lower HSZ, and restricted exposure to the CHSZ (no days), based on classification by the standard THI formula (THI = 0.8 × AT + RH% × (AT – 14.4) + 46.4), which is also demonstrated by the direction of the shift of the blue line downwards compared with the red one in Fig. 1.

**Table 4:** Heat stress modeling over the 47 study days using temperature-humidity index (THI) thresholds in natural and adaptive Buffalo housing groups

Heat Stress Zone	Natural Group (Days, THI profile range)	Adaptive Group (Days, THI range)
Critical Heat Stress Zone (CHSZ, ≥ 80.27)	23 days (80.80–83.80)	Zero days
Severe Heat Stress Zone (SHSZ, 75.40- 80.27)	18 days (75.40–79.93)	5 days (75.64–76.18)
Moderate Heat Stress Zone (MHSZ, 73.22–75.39)	5 days (73.96–74.89)	13 days (73.30–75.36)
Non-Heat Stress Zone (NHSZ, 56.71–73.21)	1 day (70.20)	29 days (65.31–73.02)

THI=0.8×AT+RH% (AT−14.4) + 46.4; Online resource 1

.

These findings statistically validate the effectiveness of environmental and housing management adaptation measures in reducing thermal stress and improving animal comfort under critical and severe heat stress conditions. **(**Table 4; Fig. 1, **and Online resource 1).**

### Heat stress, environmental adaptation, and milk production

**Fig. 2:**
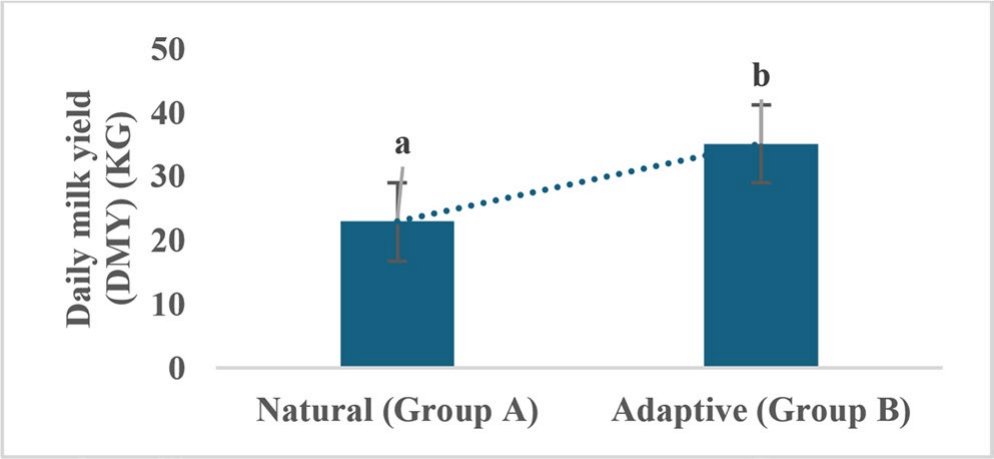
Average daily milk yield (DMY) for natural (Group **A**) and adaptive (Group **B**) buffalo Daily milk yield (DMY) represents the sum of the morning and evening yields. Values are presented as mean ± SE; Bars with different superscript letters (**a**, **b**) differ significantly at *p* < 0.05

With respect to the milk production given in Fig. 2, a notable distinction was observed every day (daily milk yield, DMY) among adaptive Group B (HS-model II) (35.15 ± 0.61 kg) and natural Group A (HS-model I) (22.94 ± 0.47 kg), which significantly improved the rate of production by 12.22 kg (53.29%) after the environmental and management interventions made in adaptive Group B (p < 0.05) (Online resource 2).

### Biometeorological statistics of heat stress (THI) and daily milk yield (DMY) as a predictive analysis for mitigating HS in buffalo

**Fig. 3:**
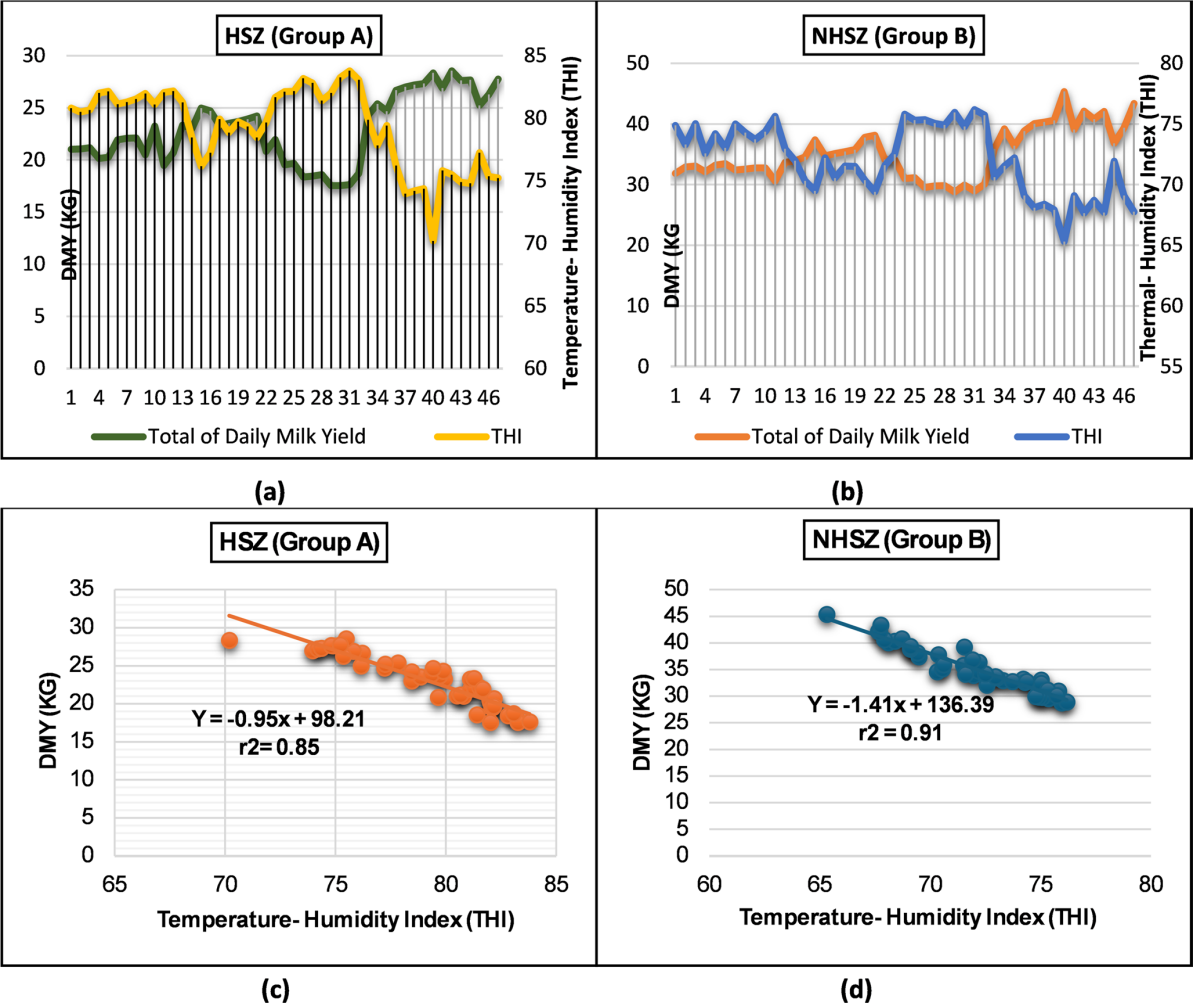
Relationship between temperature–humidity index (THI, X-axis) and daily milk yield (DMY, kg/day, Y-axis) in buffalo. (**a**, **b**) The Pearson correlation shows a strong negative association between Group A (natural) and Group B (adaptive). (**c**, **d**) Regression models with high determination coefficients (*r²* = 0.85–0.97); The data were significant at p < *0.05* THI Zone−wise and daily milk yield data and corresponding THI classifications are provided in Online Resources (1 and 2)

Pearson correlation and regression analyses were applied to evaluate the relationship between the THI and DMY in buffalo throughout the study period. A strong negative correlation was observed (r = − 0.92 and − 0.96) for Groups A and B, respectively (Fig. 3a, b), indicating that rising THI values were consistently associated with reduced milk production. Regression models confirmed these findings, with determination coefficients of r² = 0.91 (Fig. 3c) and 0.97 (Fig. 3d), both of which were statistically significant (p < 0.05). Regression analysis between THI and DMY across HS zones further revealed a consistent negative association in both natural and adaptive buffalo groups. In natural (Model I) buffalo, DMY declined by 0.23 kg/day per unit increase in THI above 69. In contrast to the adaptive (Model II) buffalo, the decline was moderated to 0.17 kg/day. Zone-wise evaluation confirmed that the lowest yields occurred in the critical HS zone (CHSZ), followed by the severe (SHSZ), moderate (MHSZ), and non-HS (NHSZ) zones, corresponding to a progressive increase in heat load. Where the DMY decreased progressively from NHSZ to CHSZ, showing the strongest negative slope in the natural exposure group (p < 0.05). These data demonstrate that adaptive management effectively mitigated production losses associated with elevated THI. In Group A (natural, Model I), this regression corresponded to an overall reduction of approximately 1.9 kg across the 70–83 THI range (from 5.3 to 3.24 kg). Conversely, in Group B (adaptive, Model II), the reduction was more minor—approximately 2.5 kg across the 65–76 THI range (from 7.49 to 4.91 kg) (Online Resource 1). These findings, statistically significant when THI exceeded 72, highlight the critical impact of HS on buffalo productivity and reinforce the value of adaptive interventions in maintaining milk performance under thermal stress.

### Impact of HS on milk quality and composition

Alongside the significant rise in daily milk yield observed in adaptive Group B, milk quality parameters showed significant improvements compared to the natural HS Group A. As indicated in Table 5, TBC of buffalo milk was significantly lower in Group B (6.13 × 10⁴ CFU/ml) than in Group A (1.15 × 10⁵ CFU/ml; p < 0.05). Similarly, SCC was markedly reduced in adaptive Group B (26.17 × 10³ cells/ml) compared with natural Group A (201 × 10³ cells/ml; p < 0.05). Furthermore, the chemical composition and physical characteristics of milk—including fat, SNF, TS, protein, salt, lactose, density, freezing point, pH, and electrical conductivity—were consistently superior in adaptive Group B relative to natural Group A.

**Table 5:** Physical, chemical, and Microbiological assessment of milk samples collected from the natural and adaptive Buffalo groups

Physical, chemical, and Microbial characteristics	Natural (Group A)	Adaptive (Group B)
TBC (CFU/ml)	1.15 × 10^5^ ± 9.94 × 10^3**a**^	6.13 × 10^4^ ± 1.23 × 10^4**b**^
SCC (n*1000/ml)	201 ± 64.07^**a**^	26.17 ± 4.16^**b**^
Fat %	5.91 ± 0.89	6.90 ± 0.28
SNF %	6.77 ± 0.15	6.89 ± 0.54
TS %	12.81 ± 1.09	13.67 ± 0.18
Protein %	3.21 ± 0.07	3.27 ± 0.03
Salt %	0.49 ± 0.01	0.50 ± 0.04
Lactose %	3.01 ± 0.07	3.07 ± 0.24
Density	20.95 ± 0.79	22.23 ± 2.13
FP (^o^C)	−0.38 ± 0.01	− 0.38 ± 0.03
Ph	6.67 ± 0.02	6.45 ± 0.10
EC	22.66 ± 0.51	23.32 ± 0.67

Data are expressed as the mean ± SEM; SNF: solid nonfat; TS: total solid; FP: freezing point; EC: electrical conductivity; Superscript letters (a, b) indicate significant differences between groups (^p<0.05);^ other data revealed non-significant differences.

### Physiological assessment of buffalo under natural HS and adaptive conditions: rectal temperature (RT) and respiratory rate (RR)

**Fig. 4:**
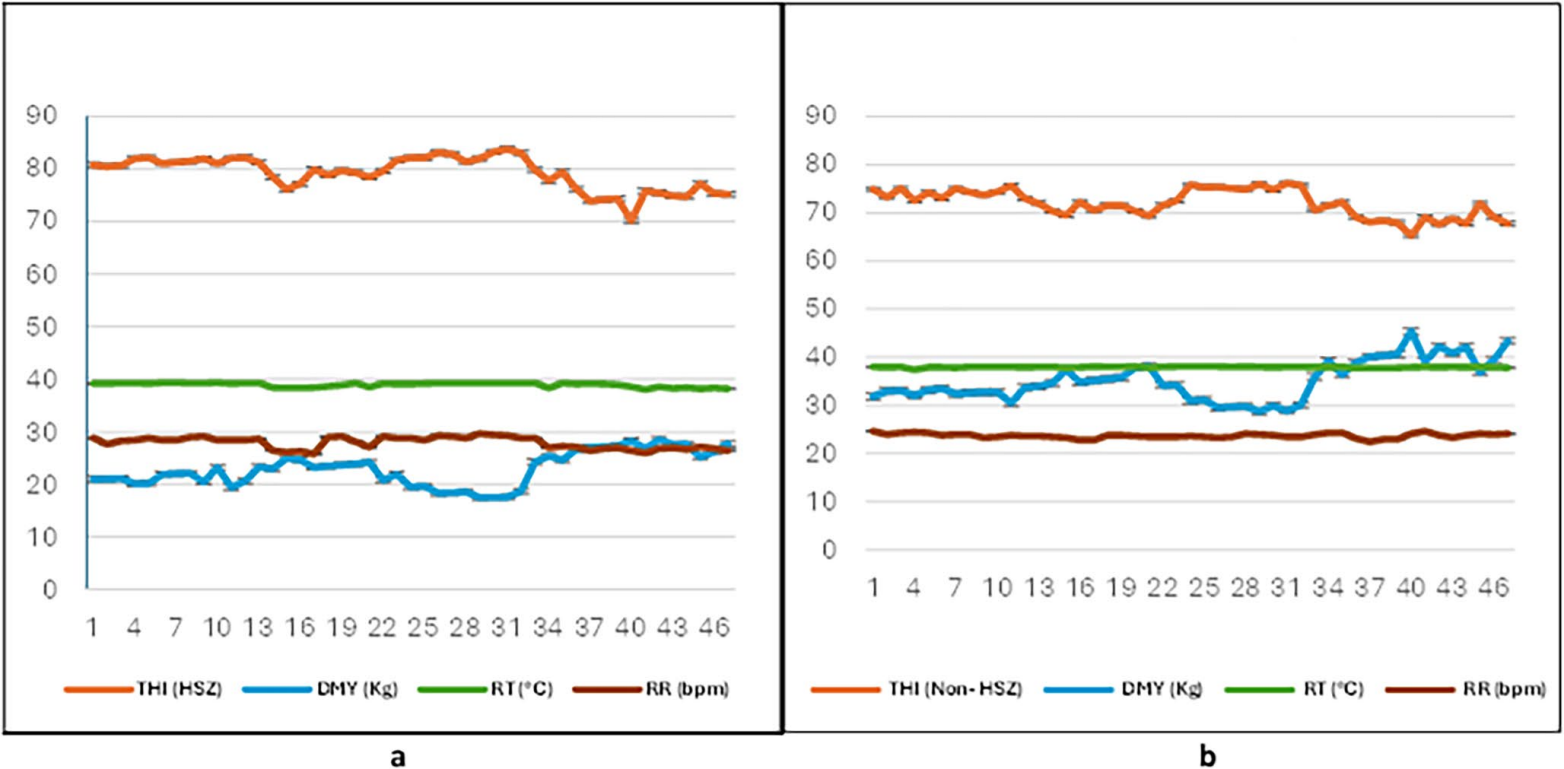
Relationship among the temperature–humidity index (THI), physiological responses, and daily milk yield (DMY) in natural and adaptive buffalo groups; (**a**) Group A (natural conditions), elevated THI was directly associated with higher rectal temperature (RT) and respiratory rate (RR), alongside reduced DMY; (**b**) Group B (adaptive conditions), THI remained below 72, corresponding to lower RT and RR values and consistently higher milk yield. Pearson correlation coefficients indicated weaker associations between THI and physiological traits in Group B (*r* = 0.38 for RT, *r* = 0.18 for RR). In contrast, Group A showed stronger responses to heat stress X-axis: THI; Y-axis: DMY (kg/day), RT (°C), and RR (bpm). Data were significant at^*p< 0.05*^ (see Online resources 3 and 4)

Heat stress had a clear impact on buffalo thermoregulation. In the natural heat-stressed (Group A), both RT and RR were significantly elevated (38.93 ± 0.01 °C and 27.96 ± 0.16 bpm, respectively) compared with the adaptive group (Group B), which showed lower values (37.91 ± 0.04 °C and 23.73 ± 0.07 bpm; *p* < 0.05) (Table 6). Correlation analysis (Fig. 4) further demonstrated that increasing THI was strongly associated with higher RT (*r* = 0.66) and RR (*r* = 0.81), both of which were negatively correlated with DMY in Group A (*r* = − 0.65 and − 0.80, respectively). By contrast, buffalo in adaptive housing (Group B), where THI remained below 72, maintained lower RT and RR alongside higher milk yield (*p* < 0.05). These findings confirm that adaptive management mitigates the physiological burden of heat stress and supports improved productivity **(Online resources 3 and 4). **

**Table 6:** Rectal temperature (RT) and respiratory rate (RR) mean values in natural and adaptive Buffalo groups during the study period

Buffaloe group	Rectal Temperature (RT) (°C)	Respiratory rate (RR) (bpm)
Group A	38.93 ± 0.01^**a**^	27.96 ± 0.16^**a**^
Group B	37.91 ± 0.04^**b**^	23.73 ± 0.07^**b**^

Data are presented as the means ± SEM; bpm (breaths per minute); different superscript letters (a&b) are significantly different at *p*< 0.05 **(Online resources 3 and 4). **

### Biochemical and molecular assessments for detecting HS in buffalo groups; tracing of oxidative stress biomarkers and transcription analysis of HS-related genes

**Fig. 5:**
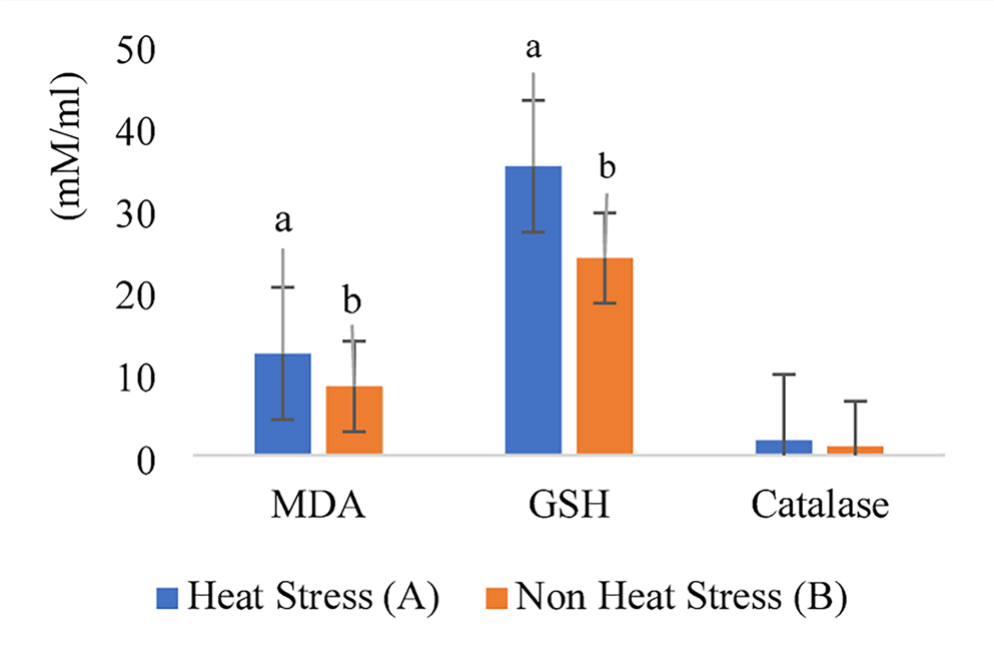
Oxidative stress biomarkers in buffalo milk: malondialdehyde (MDA), glutathione (GSH), and catalase in natural (Group **A**) and adaptive (Group **B**) buffalo groups Data are shown as means ± SEM. Bars with different superscript letters (**a**,** b**) differ significantly at^*p< 0.05*^

Milk samples from buffaloes exposed to natural heat stress (Group A) showed significantly higher levels of MDA (12.4 mM/ml) (*P* < 0.05), indicating enhanced lipid peroxidation, compared with the adaptive group (Group B, 8.4 mM/ml). Similarly, GSH and catalase activities were elevated in Group A (35.2 and 1.84 mM/mL, respectively) relative to Group B (24.0 and 1.08 mM/mL), reflecting compensatory antioxidant responses to oxidative stress. These results confirm that HS induces oxidative imbalance in buffalo milk, whereas adaptive housing mitigates this effect **(**Fig. 5).

#### Expression of energy homeostasis–related genes in buffalo milk under HS and adaptive conditions

**Fig. 6:**
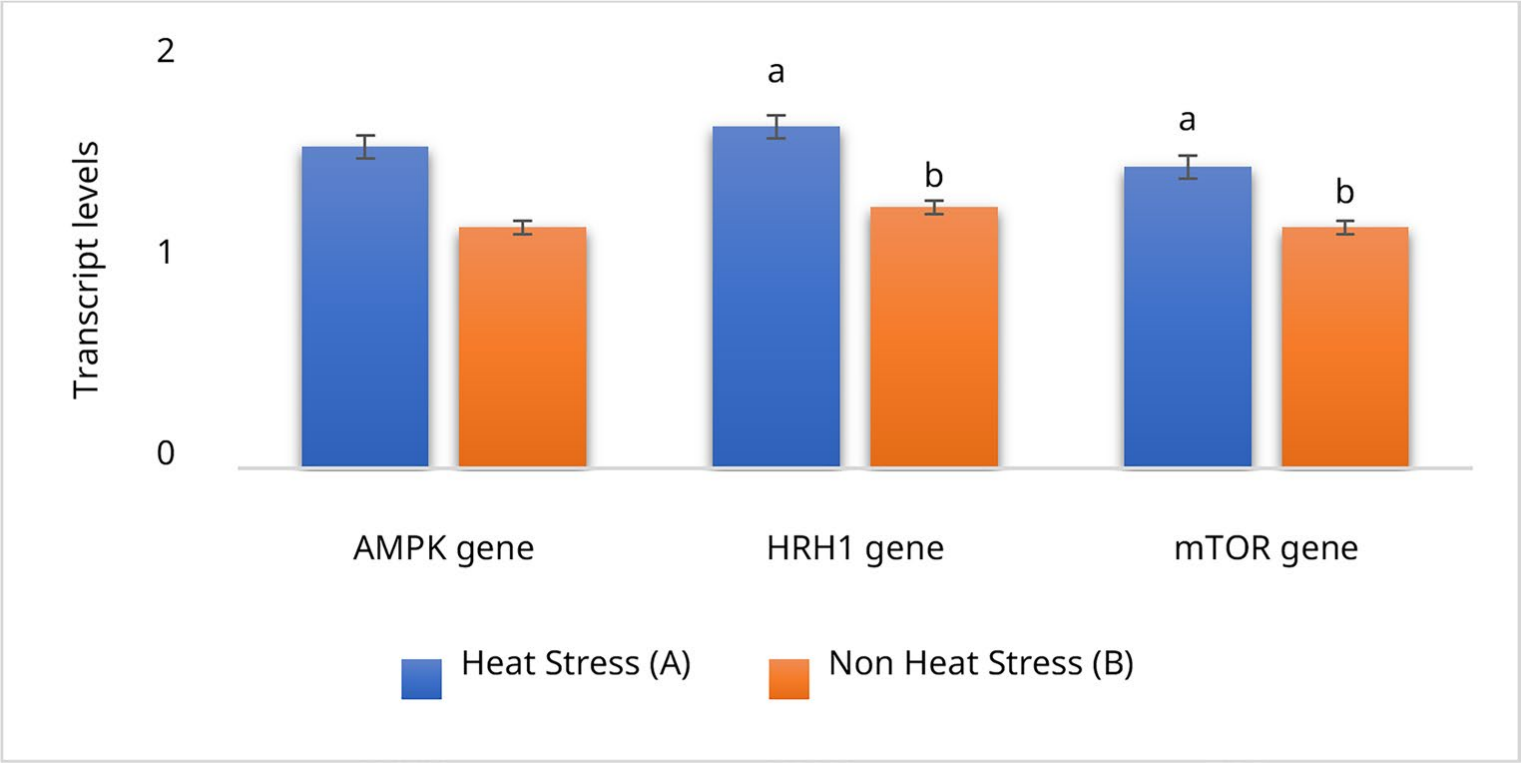
Relative transcript levels of AMPK, HRH1, and mTOR genes in buffalo milk under natural (Group **A**) and adaptive conditions (Group **B**) Data are shown as means ± SEM. Bars with different superscript letters (a, b) differ significantly at p < 0.05

As the first analysis for the transcriptional analysis of energy-regulating genes in buffalo, data in Fig. 6 revealed significantly higher expression of AMPK (1.6), HRH1 (1.7), and mTOR (1.5) in buffaloes exposed to heat stress (Group A) compared with those in the adaptive non-HS group (Group B: 1.2, 1.3, and 1.2, respectively). These findings suggest that heat stress triggers the upregulation of key genes involved in energy balance and cellular adaptation, reflecting the metabolic adjustments necessary to cope with thermal load. In contrast, adaptive housing alleviated this response, supporting physiological stability and sustained milk production.

## Discussion

This study demonstrates that adaptive environmental and managemental interventions substantially mitigate the detrimental effects of HS in lactating buffalo. Continuous monitoring of the THI provided an accurate classification of HS severity. It demonstrated that adaptive management (Model II) effectively reduced thermal load, improved physiological indicators, and enhanced milk yield and quality compared to natural exposure (Model I). It is important to note that the improved thermal comfort and productivity observed in the adaptive group reflect the cumulative benefit of multiple management interventions (ventilation, misting, wet towel evaporative cooling, and nutritional support), rather than the influence of THI reduction alone. These findings reinforce the practical value of integrating biometeorological monitoring with targeted adaptation strategies to sustain dairy buffalo productivity under subtropical climate conditions. Buffaloes represent the cornerstone of smallholder and commercial farming systems across many African and Asian countries, including Egypt, where they play a central role in milk production and rural livelihoods (Singh et al. 2015).

In this study, the highest THI values, representing critical and severe HS zones, were observed in naturally grazing buffalo during Egypt’s hot summer months of September and October (HS-Model I). Similar findings were reported by Ghavi Hossein-Zadeh et al. (2013) and Omran (2024**)**, confirming that peak summer THI coincides with maximum heat load in buffalo. Continuous THI monitoring also validated the effectiveness of adaptive environmental interventions, which alleviated HS and improved buffalo welfare and productivity. Conversely, the lowest THI values in February indicated periods of thermal comfort. However, long-term climate projections indicate a troubling trend: between 2016 and 2060, THI values in Egypt are expected to rise steadily. Khalil and Omran (2018**)** predicted that the most significant increase will occur between 2046 and 2060, during which the proportion of days within the non-HS zone is projected to decline across all Egyptian governorates, replaced by more frequent mild to moderate HS episodes. These forecasts underscore the urgent need for sustainable mitigation strategies to safeguard buffalo welfare and maintain milk production under a progressively warming climate. Dairy buffalo, although well adapted to subtropical conditions, have relatively low heat tolerance compared with other livestock species due to their dark coat and limited sweat gland density (Collier et al. 2017; Petrocchi Jasinski et al. 2023). When temperature and humidity rise above the thermal comfort zone, metabolic heat dissipation becomes inefficient, leading to HS characterized by elevated body temperature and respiratory rate. This physiological strain reduces feed intake and nutrient utilization, impairing milk synthesis. To counter these effects, adaptive environmental strategies such as shaded housing, adequate water provision, improved ventilation, and active cooling systems (fans and misters) are crucial (Omran and Fooda 2023; Khan et al. 2024; Laban et al. 2024; Slayi and Jaja 2025).

In the present study, environmental management under the adaptive model successfully reduced the THI exposure level. It shifted buffalo from critical and severe HS zones toward milder and non-HS ranges. This improvement was achieved by providing adequate barn space (10–15 m² per animal) and optimizing ventilation and cooling systems, consistent with the Guidelines for Buffalo Farming **(**Arab Organization for Agricultural Development 2022**)**. Additionally, complementary nutritional interventions, particularly electrolyte supplementation, further support thermal regulation and physiological resilience, helping buffalo maintain metabolic balance during periods of severe or critical HS. Nutritional interventions played a central role in strengthening buffalo resilience. The inclusion of multivitamins (A, D₃, E, K₃, B-complex, C), minerals (Ca, Mg, Mn, Zn, K, Na), amino acids (lysine, methionine), and probiotics enhanced antioxidant defence and supported immune function (Das et al. 2016; Nzeyimana et al. 2023; Khan et al. 2024; Verma et al. 2024). Vitamins C and E helped buffer oxidative damage, while amino acids maintained protein synthesis and repair. Milk quality parameters also improved markedly in the adaptive group. Lower TBC and SCC reflected enhanced udder health and hygiene management (Nasr 2016; Mehany et al. 2021; Farag et al. 2023). Improved milk composition, characterized by higher fat, protein, solids-not-fat, lactose, and density, corroborates earlier findings that HS negatively affects milk synthesis and composition (Bernabucci et al. 2015). Environmental and nutritional adjustments maintained metabolic efficiency and nutrient partitioning, resulting in improved milk yield and quality. These findings align with Ahmad Para et al. (2018) and Kausar and Imran (2024**)**, who reported that nutritional supplementation reduces SCC and enhances milk composition under HS.

Heat stress profoundly disrupts buffalo physiology, as evidenced by increased rectal temperature (RT) and respiratory rate (RR), both of which reduce appetite and dry matter intake, essential drivers of milk production (Polsky and von Keyserlingk 2017; Mishra 2021). In the present study, HS-Model I buffalo exhibited a significant decline in daily milk yield (DMY), a 12.22 kg or 53.29% reduction, compared with adaptive Model II buffalo. This result highlights the substantial production losses associated with HS. Research has demonstrated that THI values exceeding 75 can lead to decreased feed intake, reduced milk yield, and impaired reproductive performance in Buffalo **(**Li et al. 2020; Yan et al. 2021). The regression analysis revealed that DMY decreased by 0.17–0.23 kg/day for each unit increase in THI above 69, consistent with previous studies on cattle (Ravagnolo et al. 2000; Bouraoui et al. 2002). These results confirm THI as a reliable predictor of milk yield reduction across species, emphasizing the vulnerability of buffalo to elevated heat loads. Microclimate data from the adaptive group confirmed a consistent decrease in THI, demonstrating the cooling benefits of these interventions. Such environmental and management modifications effectively alleviated physiological strain and improved milk performance, aligning with previous reports (Baumgard and Rhoads 2013; Mishra 2021; Slayi and Jaja 2025). Overall, enhanced management practices, adequate shade and water provision, nutritional support, and upgraded barn infrastructure are critical for mitigating the impacts of HS, supporting animal welfare, and ensuring the sustainability and profitability of dairy buffalo operations.

In the context of oxidative stress, the mammary gland represents a primary site of HS-induced redox imbalance. Excessive production of reactive oxygen species (ROS) disrupts mammary epithelial cell integrity, impairs milk biosynthesis, and triggers compensatory antioxidant responses. In this study, heat-stressed buffalo exhibited elevated MDA levels, accompanied by increased GSH and catalase activity, reflecting an increased oxidative load and activation of cellular defence mechanisms. These findings align with previous reports showing that HS negatively affects milk yield and physiological performance (Bouraoui et al. 2002) and induces oxidative stress responses in lactating animals (Nandi et al. 2019). At the mammary level, oxidative stress alters the milk antioxidant profile, which mirrors the udder’s oxidative status and serves as a valuable non-invasive biomarker for assessing heat load. Prior studies have confirmed that HS elevates ROS, lipid peroxides, and nitric oxide levels, along with enhanced activities of antioxidant enzymes such as superoxide dismutase, catalase, glutathione peroxidase, and glutathione reductase (Waiz et al. 2016; Hady et al. 2018; Nandi et al. 2019; Sharma et al. 2023). The observed upregulation of GSH and catalase in the present study suggests a compensatory mechanism that helps limit oxidative damage; however, this response may become insufficient under prolonged or severe HS exposure, ultimately overwhelming the animal’s physiological buffering capacity (Bawish et al. 2023; Khan et al. 2024).

Building on these physiological insights, molecular exploration offers a deeper understanding of how buffalo respond to thermal load. This study provides novel insights into gene expression changes associated with HS adaptation in Egyptian buffalo. The observed upregulation of *AMPK*, *HRH1*, and *mTOR* genes in the natural exposure group (Model I) suggests the activation of energy homeostasis pathways as a compensatory mechanism to counteract thermal load. Increased *AMPK* expression likely reflects an attempt to restore cellular energy balance under reduced feed intake and altered metabolism. At the same time, *HRH1* upregulation indicates modulation of the immune–inflammatory response during oxidative stress. Similarly, elevated *mTOR* transcription denotes heightened anabolic demand required for metabolic adaptation under prolonged HS (Oakhill et al. 2010; Kakigi et al. 2011; Shimamura et al. 2011; Lipton and Sahin 2014; Yu et al. 2018; Liang et al. 2020; Huang et al. 2021; Nagai and Kaji 2023).

Interestingly, Kakigi et al. (2011) reported that HS enhances *mTOR* signaling following resistance exercise in human skeletal muscle, suggesting a conserved stress–metabolism link across species. In contrast, Huang et al. (2021) observed downregulation of this pathway in another context, reflecting possible species- or condition-specific differences in gene regulation. Taken together, our findings in buffalo underscore that the differential expression of *AMPK*, *HRH1*, and *mTOR* reflects a coordinated molecular adaptation to manage energy metabolism under HS. These insights deepen our understanding of buffalo resilience and highlight the potential for genetic selection and targeted research to develop heat-tolerant breeds that can sustain productivity in the face of climate change.

Although a detailed cost–benefit analysis was beyond the scope of the current work, the marked improvement in milk yield and quality under adaptive management indicates clear economic potential. Implementing adaptive interventions—such as improved housing, nutritional supplementation, and enhanced thermal regulation—may offset their initial costs through gains in productivity and animal welfare.

In conclusion, HS poses a significant constraint on dairy buffalo production amid global climate change, with substantial implications for milk yield, composition, and overall animal welfare. This study demonstrates that adaptive housing, optimized nutrition, and improved management practices can effectively mitigate these negative impacts. The integration of physiological, biochemical, and molecular evidence highlights how adaptive interventions mitigate thermal stress, enhance milk production, and maintain energy homeostasis. Collectively, these findings underscore the need for a comprehensive resilience framework that combines environmental control, nutritional optimization, regular health monitoring, and long-term genetic selection to safeguard the productivity and welfare of buffalo herds in regions experiencing HS.

## Supplementary Information

Below is the link to the electronic supplementary material.

Supplementary Material 1 (PDF 502 KB)

## Data Availability

All the data produced or analyzed in this study are provided within this published article or available in the [ESM_1.pdf] repository, supplementary information file (Online resources 1-4). Additional data can be accessed on the department’s shared drive or obtained from the corresponding author upon reasonable request.

## References

[CR1] Abdel-Gawad DRI, Ibrahim MA, Moawad UK, Kamel S, El-Banna HA, Hassan WH, El-Ela FIA (2023) Effectiveness of natural biomaterials in the protection and healing of experimentally induced gastric mucosa ulcer in rats. Mol Biol Rep 50:9085–9098. 10.1007/s11033-023-08776-9 37741810 10.1007/s11033-023-08776-9 PMC10635934

[CR2] Aebi H (1984) Catalase in vitro. Methods Enzymol 105:121–126 6727660 10.1016/s0076-6879(84)05016-3

[CR3] Ahmad Para I, Ahmad Dar P, Ahmad Malla B et al (2018) Impact of heat stress on the reproduction of farm animals and strategies to ameliorate it. Biol Rhythm Res 51(4):616–632. 10.1080/09291016.2018.1548870

[CR4] American Society of Heating, Refrigerating and Air-Conditioning Engineers (ASHRAE) (2019) HVAC Applications: Chap. 24 – Agricultural Facilities, Atlanta, GA, USA. Available at: https://www.ashrae.org/technical-resources/ashrae-handbook

[CR5] Arab Organization for Agricultural Development (2022) Guidelines for Buffalo farming in the Arab world and beyond. Khartoum, Sudan. https://www.aoad.org/Baf.Guidlines2022.pdf

[CR6] Arefaine H, Kashwa MA (2015) Review on strategies for sustainable Buffalo milk production in Egypt. J Biol Agricult Healthc 5:63–67

[CR7] Baumgard LH, Rhoads RP (2013) Effects of heat stress on postabsorptive metabolism and energetics. Annu Rev Anim Biosci 1:311–337. 10.1146/annurev-animal-031412-103644 25387022 10.1146/annurev-animal-031412-103644

[CR8] Bawish BM, Rabab MA, Gohari ST, Khattab MH, Abdelkader NA, Elsharkawy SH et al (2023) Promotion effect of *Geranium robertianum* L. leaves and Aloe Vera gel powder on Aspirin^®^-induced gastric ulcers in Wistar rats: anxiolytic behavioral effect, antioxidant activity, and protective pathways. Inflammopharmacology 31:3183–3201. 10.1007/s10787-023-01205-0 37184667 10.1007/s10787-023-01205-0 PMC10692037

[CR9] Behera R, Chakravarty AK, Kashyap N, Sahu A, Deshmukh B, Dash S (2023) Heat index-based identification of critical heat stress zone for production traits in Murrah Buffalo under subtropical climate. Biol Rhythm Res 54:334–343. 10.1080/09291016.2023.2171225

[CR10] Berman A, Horovitz T, Kaim M, Gacitua H (2016) A comparison of THI indices leads to a sensible heat-based heat stress index for shaded cattle that aligns temperature and humidity stress. Int J Biometeorol 60(10):1453–1462 26817655 10.1007/s00484-016-1136-9

[CR11] Bernabucci U, Basiricò L, Morera P, Dipasquale D, Vitali A, Piccioli Cappelli F, Calamari L (2015) Effect of summer season on milk protein fractions in Holstein cows. J Dairy Sci 98(3):1815–1827. 10.3168/jds.2014-8788 25547301 10.3168/jds.2014-8788

[CR12] Biomath (2023) Power and sample size calculation for t-tests. Retrieved from http://www.biomath.info/power/ttest.htm, accessed August 2023

[CR13] Bouraoui R, Lahmar M, Majdoub A, Djemali M, Belyea R (2002) The relationship of temperature-humidity index with milk production of dairy cows in a mediterranean climate. Anim Res 51(6):479–491. 10.1051/animres:2002036

[CR14] Collier RJ, Renquist BJ, Xiao YA (2017) A 100-year review: stress physiology including heat stress. J Dairy Sci 102(12):10367–10380. 10.3168/jds.2017-13676 10.3168/jds.2017-13676 29153170

[CR15] Das R, Sailo L, Verma N, Bharti P, Saikia J, Imtiwati, Kumar R (2016) Impact of heat stress on health and performance of dairy animals: a review. Vet World 9:260–268. 10.14202/vetworld.2016.260-268 27057109 10.14202/vetworld.2016.260-268 PMC4823286

[CR16] Dash S, Chakravarty AK, Singh A, Behera R, Upadhyay A, Shivahre PR (2014) Determination of critical heat stress zone for fertility traits using temperature humidity index in Murrah buffaloes. Indian J Anim Sci 84(11):1181–1184

[CR17] Dash S, Chakravarty AK, Sah V, Jamuna V, Behera R, Kashyap N, Deshmukh B (2015) Influence of temperature and humidity on pregnancy rate of Murrah Buffalo. Asian-Aust J Anim Sci 28:943–950. 10.5713/ajas.14.0825 10.5713/ajas.14.0825 PMC4478503 26104398

[CR18] Dash S, Chakravarty AK, Singh A, Upadhyay A, Singh M, Yousuf S (2016) Effect of heat stress on reproductive performances of dairy cattle and buffaloes: a review. Vet World 9(3):235–244. 10.14202/vetworld.2016.235-244 27057105 10.14202/vetworld.2016.235-244 PMC4823282

[CR19] Ellman GL (1959) Tissue sulfhydryl groups. Arch Biochem Biophys 82:70–77 13650640 10.1016/0003-9861(59)90090-6

[CR20] Fahim KM, Ismael E, Khalefa HS, Farag HS, Hamza DA (2019) Isolation and characterization of *E. coli* strains causing intramammary infections from dairy animals and wild birds. Int J Vet Sci Med 7:61–70. 10.1080/23144599.2019.1691378 31840026 10.1080/23144599.2019.1691378 PMC6896447

[CR21] FAO FAOSTAT (2022) Production Yield quantities of buffalo raw milk in Egypt (2012–2022). Accessed 20th December 2024. https://www.fao.org/faostat/en/#data/QCL/visualize

[CR22] Farag HS, Sharif SA, Fahim KM, Fayed AA, Abdelfattah EM, El-Sayed SM, Hegazy YM, ElAshmawy WR (2023) Management practices of bovine mastitis and milk quality on Egyptian dairies. Vet Sci 10:629. 10.3390/vetsci10100629 37888581 10.3390/vetsci10100629 PMC10611314

[CR23] Ghavi Hossein-Zadeh N, Mohit A, Azad N (2013) Effect of temperature-humidity index on productive and reproductive performances of Iranian Holstein cows. Iran J Vet Res 14:106–112

[CR24] Hady MM, Melegy TM, Anwar SR (2018) Impact of the Egyptian summer season on oxidative stress biomarkers and some physiological parameters in crossbred cows and Egyptian Buffalo. Vet World 11:771–778. 10.14202/vetworld.2018.771-777 30034168 10.14202/vetworld.2018.771-778 PMC6048079

[CR25] Hassanen EI et al (2024) Phenolic-rich fraction of green tea attenuates histamine-mediated cardiopulmonary toxicity by inhibiting Cox-2/NF-κB signaling pathway and regulating oxidant/antioxidant balance. Beni-Suef Univ J Basic Appl Sci 13:6. 10.1186/s43088-024-00464-2

[CR26] Huang Y, Xie H, Pan P et al (2021) Heat stress promotes lipid accumulation by inhibiting the AMPK-PGC-1α signaling pathway in 3T3-L1 preadipocytes. Cell Stress Chaperones 26:563–574. 10.1007/s12192-021-01201-9 33743152 10.1007/s12192-021-01201-9 PMC8065074

[CR27] International Organization for Standardization (ISO) (1998) ISO 7726: Ergonomics of the thermal environment – Instruments for measuring physical quantities. International Organization for Standardization, Geneva, Switzerland. Available at: https://www.iso.org/standard/14567.html

[CR28] International Organization for Standardization (ISO) (2013) ISO 4833-2:2013 Microbiology of the food chain — Horizontal method for the enumeration of microorganisms — Part 2: Colony count at 30°C by the surface plating technique. https://cdn.standards.iteh.ai/samples/59509/d4ebb86a3ceb480eb4ff5248b9e9867b/ISO-4833-2-2013.pdf

[CR29] Kakigi R, Naito H, Ogura Y, Kobayashi H et al (2011) Heat stress enhances mTOR signaling after resistance exercise in human skeletal muscle. J Physiol Sci 61:131–140. 10.1007/s12576-010-0130-y 21222186 10.1007/s12576-010-0130-y PMC10717825

[CR72] Kamel S, Ibrahim MA, Awad ET, El-Hindi HMA, Abdel-Aziz SA (2018). Molecular cloning and characterization of the novel CYP2J2 in dromedary camels (Camelus dromedarius). Int J Biol Macromol 120(Pt B):1770–1776. 10.1016/j.ijbiomac.2018.09.193 10.1016/j.ijbiomac.2018.09.193 30287372

[CR30] Kausar R, Imran S (2024) Heat Stress Mitigation through Feeding and Nutritional Interventions in Ruminants. IntechOpen. in Babinszky L (Ed.) Latest Scientific Findings in Ruminant Nutrition - Research for Practical Implementation. IntechOpen. 10.5772/intechopen.1005594

[CR31] Khalil AA, Omran FI (2018) Impact of climate change on temperature humidity index values in Egypt. Int J Sci Res Sci Eng Technol 4:1059–1064

[CR32] Khan MZ, Khan A, Chen W, Chai W, Wang C (2024) Advancements in genetic biomarkers and exogenous antioxidant supplementation for safeguarding mammalian cells against heat-induced oxidative stress and apoptosis. Antioxidants 13:258. 10.3390/antiox13030258 38539792 10.3390/antiox13030258 PMC10967571

[CR33] Laban SE, Elhady RH, Zaki MM et al (2024) Evaluation of biosecurity practices applied on some dairy cattle farms in Egypt and their impact on milk quality and production. J Adv Vet Res 14:1026–1032. https://advetresearch.com/index.php/AVR/article/view/1932

[CR34] Li G, Chen S, Chen J, Peng D, Gu X (2020) Predicting rectal temperature and respiration rate responses in lactating dairy cows exposed to heat stress. J Dairy Sci 103:5466–5484. 10.3168/jds.2019-16411 32278558 10.3168/jds.2019-16411

[CR35] Liang Y, Tabler GT, Dridi S (2020) Sprinkler technology improves broiler production sustainability: from stress alleviation to water usage conservation. Front Vet Sci 7:544814. 10.3389/fvets.2020.544814 33195519 10.3389/fvets.2020.544814 PMC7536280

[CR36] Lipton JO, Sahin M (2014) The neurology of mTOR. Neuron 84:275–291. 10.1016/j.neuron.2014.09.034 25374355 10.1016/j.neuron.2014.09.034 PMC4223653

[CR37] Liu Z, Ezernieks V, Wang J et al (2017) Heat stress in dairy cattle alters lipid composition of milk. Sci Rep 7:961. 10.1038/s41598-017-01120-9 28424507 10.1038/s41598-017-01120-9 PMC5430412

[CR38] Livak KJ, Schmittgen HD (2001) Analysis of relative gene expression data using Real-Time quantitative PCR and the 2–2∆∆CT method. Methods 25:402–408 11846609 10.1006/meth.2001.1262

[CR39] Mader TL, Davis MS, Brown-Brandl T (2006) Environmental factors influencing heat stress in feedlot cattle. J Anim Sci 84(3):712–719. 10.2527/2006.843712x 16478964 10.2527/2006.843712x

[CR40] Marai IFM, Habeeb AAM (2010) Buffalo’s biological functions as affected by heat stress – a review. Livest Sci 127:89–109. 10.1016/j.livsci.2009.08.001

[CR41] Mehany EN, El-Shafaie MA, Fahim KM, Sallam SS (2021) Relationship between somatic cell count and different microbial and chemical quality parameters of bulk tank milk. Adv Anim Vet Sci 9:1660–1668. 10.17582/journal.aavs/2021/9.10.1660.1668

[CR42] Minervino AHH, Zava M, Vecchio D, Borghese A (2020) *Bubalus bubalis*: a short story. Front Vet Sci 7:570413. 10.3389/fvets.2020.570413 33335917 10.3389/fvets.2020.570413 PMC7736047

[CR43] Mishra SR (2021) Thermoregulatory responses in riverine buffalo against heat stress: an updated review. J Thermobiol 96:102844. 10.1016/j.jtherbio.2021.102844 10.1016/j.jtherbio.2021.102844 33627281

[CR44] Mohamed SM, Shalaby MA, El-Shiekh RA, Bakr AF et al (2024) Maca roots: a potential therapeutic in the management of metabolic disorders through the modulation of metabolic biochemical markers in rats fed high-fat high-carbohydrate diet. J Ethnopharmacol 321:117533. 10.1016/j.jep.2023.117533 38056538 10.1016/j.jep.2023.117533

[CR45] Moran J (2005) Tropical Dairy Farming: Feeding Management for Smallholder Dairy Farmers in the Humid Tropics. Landlinks Press, Collingwood. 10.1071/9780643093133

[CR46] Nagai M, Kaji H (2023) Thermal effect on heat shock protein 70 family to prevent atherosclerotic cardiovascular disease. Biomolecules 13:867. 10.3390/biom13050867 37238736 10.3390/biom13050867 PMC10216495

[CR47] Nandi A, Yan LJ, Jana CK, Das N (2019) Role of catalase in oxidative stress- and age-associated degenerative diseases. Oxid Med Cell Longev 9613090. 10.1155/2019/9613090 31827713 10.1155/2019/9613090 PMC6885225

[CR48] Nasr MA (2016) The impact of crossbreeding Egyptian and Italian Buffalo on milk yield and composition under subtropical environmental conditions. J Dairy Res 83:196–201. 10.1017/S0022029916000194 27210493 10.1017/S0022029916000194

[CR49] National Research Council (NRC) (1971) A guide to environmental research on animals. National Academy of Sciences, Washington, DC, USA

[CR50] Nzeyimana JB, Fan C, Zhuo Z, Butore J, Cheng J (2023) Heat stress effects on the lactation performance, reproduction, and alleviating nutritional strategies in dairy cattle, a review. J Anim Behav Biometeorol 11(3):2023018. 10.31893/jabb.23018

[CR51] Oakhill JS, Oakhill S, Chen Z, Scott JW et al (2010) β-Subunit myristoylation is the gatekeeper for initiating metabolic stress sensing by AMP-activated protein kinase (AMPK). Proc Natl Acad Sci USA 107:19237–19241. 10.1073/pnas.1009705107 20974912 10.1073/pnas.1009705107 PMC2984171

[CR52] Ohkawa H, Ohishi N, Yagi K (1979) Assay for lipid peroxides in animal tissues by thiobarbituric acid reaction. Anal Biochem 95:351–358. 10.1016/0003-2697(79)90738-3 36810 10.1016/0003-2697(79)90738-3

[CR53] Omran F (2024) Climatic challenges for domestic ruminants under the Egyptian conditions in relation to temperature humidity index (THI). Egypt J Agric Res 102:493–507. 10.21608/ejar.2024.270277.1516

[CR54] Omran FI, Fooda TA (2023) Impact of chronic heat stress on physiological and productive performance of Egyptian Buffalo. Egypt J Agric Res 101:151–165. https://ejar.journals.ekb.eg/article_284241.html

[CR55] Omran FI, Khali AA, Fooda T (2020) Physiological responses and hematological aspects of Buffalo and cows under different Climatic conditions in Egypt. Egypt J Agric Res 98:64–79. 10.21608/ejar.2020.101425

[CR56] Petrocchi Jasinski F, Evangelista C, Basiricò L, Bernabucci U (2023) Responses of dairy Buffalo to heat stress conditions and mitigation strategies: a review. Animals 13:1260. 10.3390/ani13071260 37048516 10.3390/ani13071260 PMC10093017

[CR57] Polsky L, von Keyserlingk MAG (2017) Invited review: effects of heat stress on dairy cattle welfare. J Dairy Sci 100(11):8645–8657. 10.3168/jds.2017-12651 28918147 10.3168/jds.2017-12651

[CR58] Purohit PB, Gupta JP, Chaudhri JD, Bhatt TM, Pawar M, Srivastava A, Patel MP (2020) Effect of heat stress on production and reproduction potential of dairy animals vis-à-vis Buffalo. Int J Livest Res 10:3

[CR59] Rabie TSKM (2020) Potential climate change impacts on livestock and food security nexus in Egypt. In: Ewis Omran, ES., Negm, A. (eds) Climate Change Impacts on Agriculture and Food Security in Egypt. Springer Water. Springer, Cham. pp 420–450. 10.1007/978-3-030-41629-4_17

[CR60] Ravagnolo O, Misztal I, Hoogenboom G (2000) Genetic component of heat stress in dairy cattle, development of heat index function. J Dairy Sci 83:2120–2125. 10.3168/jds.S0022-0302(00)75094-6 11003246 10.3168/jds.S0022-0302(00)75094-6

[CR61] Sharma S, Sharma V, Konwar D, Khan A, Kumar D, Brahma B (2023) A comparative study on effect of heat stress on physiological and cellular responses of crossbred cattle and riverine Buffalo in subtropical region of India. Int J Biometeorol 67(10):1619–1628. 10.1007/s00484-023-02523-2 37495744 10.1007/s00484-023-02523-2

[CR62] Shimamura T, Shiroishi M, Weyand S et al (2011) Structure of the human Histamine H1 receptor complex with Doxepin. Nature 475:65–70. 10.1038/nature10236 21697825 10.1038/nature10236 PMC3131495

[CR63] Singh B, Gautam SK, Chauhan MS, Singla SK (2015) Textbook of Animal Biotechnology. The Energy and Resources Institute (TERI). New Delhi, India: 1st edn

[CR64] Slayi M, Jaja IF (2025) Strategies for mitigating heat stress and their effects on behavior, physiological indicators, and growth performance in communally managed feedlot cattle. Front Vet Sci. 10.3389/fvets.2025.1513368 40027359 10.3389/fvets.2025.1513368 PMC11868087

[CR65] Smith LG, Kirk GJ, Jones PJ, Williams AG (2019) The greenhouse gas impacts of converting food production in England and Wales to organic methods. Nat Commun 10(1):1–10. 10.1038/s41467-019-12622-7 31641128 10.1038/s41467-019-12622-7 PMC6805889

[CR66] Verma P, Jain AK, Mishra A, Jesse DD, Mandal S, Gattani A, Patel P, Singh P, Jatav M (2024) Ameliorative effect of Withania somnifera on haemato-biochemical and hormonal parameters during heat stress in Murrah buffalo calves. Int J Adv Biochem Res 8(4S):80–85. 10.33545/26174693.2024.v8.i4Sb.910

[CR67] Waiz SA, Raies-Ul-Haq M, Dhanda S, Kumar A, Goud TS, Chauhan MS, Upadhyay RC (2016) Heat stress and antioxidant enzyme activity in Bubaline (*Bubalus bubalis*) oocytes during in vitro maturation. Int J Biometeorol 60:1357–1366. 10.1007/s00484-015-1129-0 26781547 10.1007/s00484-015-1129-0

[CR68] World Meteorological Organisation (WMO) (2023) Banha Station, Egypt (WMO ID IBANHA3) weather conditions and climate. Weather Underground. Retrieved September and October 2023 https://www.wunderground.com/dashboard/pws/IBANHA3

[CR69] Yan G, Liu K, Hao Z, Shi Z, Li H (2021) The effects of cow-related factors on rectal temperature, respiration rate, and temperature-humidity index thresholds for lactating cows exposed to heat stress. J Therm Biol 100:103041. 10.1016/j.jtherbio.2021.103041 34503788 10.1016/j.jtherbio.2021.103041

[CR70] Yu CL, Guan J, Ding J, Huang S, Lian Y, Luo H, Wang X (2018) AMP-activated protein kinase negatively regulates heat treatment-induced lactate secretion in cultured Boar Sertoli cells. Theriogenology 121:35–41. 10.1016/j.theriogenology.2018.07.039 30125826 10.1016/j.theriogenology.2018.07.039

[CR71] Zhang J, Jeong KS (2022) Evaluating the impact of heat stress on milk quality in South Korea. Anim Prod Sci 62:1501–1506. 10.1071/AN21592

