# Supplementary material for: Heat stress in dairy buffalo: biometeorological, molecular, and adaptive strategies for climate change resilience in subtropical regions
Source: Vet Res Commun. 2026 Jan 16;50(2):107. doi: 10.1007/s11259-025-11009-y (PMC12811276; doi:10.1007/s11259-025-11009-y)
Supplement: Supplementary file 1 — Supplementary Material 1 (PDF 502 KB) [file 11259_2025_11009_MOESM1_ESM.pdf]

Supplementary information for the *Journal of Veterinary Research Communications*

Heat Stress in Dairy Buffalo: Biometeorological, Molecular, and Adaptive Strategies for Climate Change Resilience in Subtropical Regions

Eman M. Ismail[1*], Aly M. Aly[2], Heba S. Farag[3], Shaimaa Kamel[4], and Karima M. Fahim[5]

[1*] Department of Veterinary Hygiene and Management, Faculty of Veterinary Medicine, Cairo University, Giza 12211, Egypt;[*]dr_eman252@cu.edu.eg, ID-0000-0001-6191-6950.

Online Resource 1. Meteorological data from 1st Sept to 17th Oct 2023, study duration for the natural group (Group A) and adaptive group (Group B)

Experiment day	Day/M	Ambient temperature (°C)		Outdoor DP (°C)	RH (%)		THI	
		Outdoor (Ta, °C)	Indoor (Tb, °C)		Outdoor	Indoor	Outdoor	Indoor
1	1st Sept	33	28	18.77	43.02	44.76	80.80 [a]	74.89 [C]
2	2nd Sept	33	27	18.6	41.41	42.06	80.50 [a]	73.30 [C]
3	3rd Sept	33	27	18.49	42.28	55.73	80.66 [a]	75.02 [C]
4	4th Sept	34	28	19.64	42.94	27.70	82.02 [a]	72.57 d
5	5th Sept	34	27	19.88	43.59	49.26	82.14 [a]	74.21 [C]
6	6th Sept	33	27	19.39	44.72	39.83	81.12 [a]	73.02 [d]
7	7th Sept	33	28	19.73	45.67	45.72	81.30 [a]	75.02 [C]
8	8th Sept	33	28	20.18	46.96	40.43	81.53 [a]	74.30 [C]
9	9th Sept	34	28	19.57	42.76	36.21	81.98 [a]	73.72 [C]
10	10th Sept	33	28	19.36	44.63	40.93	81.10 [a]	74.37 [C]
11	11th Sept	35	29	18.23	37.20	41.39	82.06 [a]	75.64 [b]
12	12th Sept	34	27	19.94	43.75	39.29	82.18 [a]	72.95 [d]
13	13th Sept	33	27	19.69	45.56	31.44	81.27 [a]	71.96 [d]
14	14th Sept	32	26	15.34	36.63	26.89	78.45 [b]	70.32 [d]
15	15th Sept	30	25	13.69	36.91	29.08	76.16 [b]	69.48 [d]
16	16th Sept	31	26	14.32	36.31	43.18	77.23 [b]	72.21 [d]
17	17th Sept	32	26	18.6	45.04	28.82	79.93 [b]	70.54 [d]
18	18th Sept	32	26	16.3	38.95	37.43	78.86 [b]	71.54 [d]
19	19th Sept	32	26	18.17	43.84	37.11	79.72 [b]	71.50 [d]
20	20th Sept	32	25	17.38	41.71	37.50	79.34 [b]	70.38 [d]
21	21st Sept	32	25	15.35	36.66	28.46	78.45 [b]	69.42 [d]
22	22nd Sept	34	27	14.44	30.90	28.54	79.66 [b]	71.60 [d]
23	23rd Sept	34	27	18.96	41.17	35.74	81.67 [a]	72.50 [d]
24	24th Sept	35	29	18.36	37.50	42.56	82.13 [a]	75.81 [b]
25	25th Sept	35	29	18.43	37.67	39.13	82.16 [a]	75.31 [C]
26	26th Sept	36	30	19.00	36.94	31.78	83.18 [a]	75.36 [C]
27	27th Sept	36	30	18.26	35.27	29.92	82.82 [a]	75.07 [C]
28	28th Sept	36	30	15.04	28.74	28.94	81.41 [a]	74.91 [C]
29	29th Sept	37	31	14.68	26.59	28.63	82.01 [a]	75.95 [b]
30	30th Sept	37	30	17.65	32.13	28.08	83.26 [a]	74.78 [C]
31	1st Oct	37	31	18.77	34.47	30.01	83.80 [a]	76.18 [b]
32	2nd Oct	36	30	18.60	36.41	34.19	83.06 [a]	75.73 [b]
33	3rd Oct	32	26	18.49	44.73	29.36	79.87 [b]	70.61 [d]
34	4th Oct	31	26	15.79	39.91	37.41	77.83 [b]	71.54 [d]
35	5th Oct	32	26	17.54	42.14	43.18	79.42 [b]	72.21 [d]
36	6th Oct	29	24	15.98	45.32	36.44	76.22 [b]	69.10 [d]
37	7th Oct	28	23	12.33	37.92	38.44	73.96 [C]	68.11 [d]
38	8th Oct	28	23	13.02	39.67	41.86	74.20 [C]	68.40 [d]

39	9th Oct	28	23	13.50	40.93	36.33	**74.37 C**	**67.92 d**
40	10th Oct	23	20	15.50	62.68	51.95	**70.20 d**	**65.31 d**
41	11th Oct	29	24	15.00	42.56	36.44	**75.81 b**	**69.10 d**
42	12th Oct	28	22	16.35	49.17	48.04	**75.49 b**	**67.65 d**
43	13th Oct	28	23	14.88	44.76	45.51	**74.89 C**	**68.71 d**
44	14th Oct	28	22	14.65	44.10	48.78	**74.80 C**	**67.71 d**
45	15th Oct	32	26	12.16	29.80	40.72	**77.24 b**	**71.92 d**
46	16th Oct	30	24	11.41	31.78	36.44	**75.40 b**	**69.10 d**
47	17th Oct	28	22	15.71	47.21	49.50	**75.22 b**	**67.76 d**
Mean ±SE		32.23 ± 0.44	26.43 ± 0.38	16.92± 0.35	40.46 ± 0.90	37.90 ± 1.07	**79.30 b ± 0.47**	**72.02 d ±0.42**

Ta (°C): Outdoor ambient temperature,(DP (°C): Dew Point temperature (°C), RH (%): Relative Humidity %, Tb (°C): Indoor barn temperature (°C), THI: Temperature Humidity Index; Heat stress zone categorization: a CHSZ (≥ 80.28), b SHSZ (75.40-80.27), c MHSZ (73.22-75.39), d (NHSZ (56.71-73.21)

Online resource 2. Total and average Daily Milk production data sheets from 1st Sept to 17th Oct 2023, study duration for each buffalo of the natural (Gp A) and adaptive group (Gp B)

No.	Day/month	Daily Milk Yield/buffalo of Gp A						TDMY Gp A (A1-A6)	Daily Milk Yield/buffalo of Gp B						TDMY Gp B (B1-B6)
		A1	A2	A3	A4	A5	A6		B1	B2	B3	B4	B5	B6	
1	1st Sept	2.68	3.80	3.07	3.11	4.67	3.69	**21.02**	6.25	6.32	4.66	4.89	4.92	4.81	**31.85**
2	2nd Sept	2.68	3.80	3.10	3.12	4.67	3.69	**21.06**	6.75	6.70	4.26	5.10	5.13	4.97	**32.91**
3	3rd Sept	2.71	3.85	3.13	3.13	4.67	3.69	**21.18**	6.85	6.70	4.28	5.12	5.13	4.97	**33.05**
4	4th Sept	2.55	3.27	3.45	2.87	4.54	3.47	**20.15**	6.35	6.40	4.69	4.90	4.95	4.81	**32.10**
5	5th Sept	2.58	3.33	3.45	2.88	4.54	3.47	**20.25**	6.97	6.70	4.29	5.12	5.13	4.99	**33.20**
6	6th Sept	2.75	3.89	3.61	3.22	4.75	3.72	**21.94**	7.05	6.72	4.32	5.20	5.15	5.03	**33.47**
7	7th Sept	2.75	3.92	3.62	3.28	4.75	3.75	**22.07**	6.50	6.45	4.69	4.94	5.00	4.82	**32.40**
8	8th Sept	2.75	3.97	3.62	3.28	4.75	3.75	**22.12**	6.60	6.45	4.69	4.97	5.00	4.87	**32.58**
9	9th Sept	2.58	3.40	3.50	2.94	4.54	3.53	**20.49**	6.67	6.45	4.72	5.00	5.01	4.9	**32.75**
10	10th Sept	2.76	4.00	3.63	3.32	4.75	4.78	**23.24**	6.67	6.45	4.74	5.00	5.01	4.9	**32.77**
11	11th Sept	2.45	3.20	3.35	2.60	4.47	3.49	**19.47**	5.60	6.13	4.50	4.72	4.86	4.77	**30.58**
12	12th Sept	2.61	3.45	3.53	2.95	4.60	3.55	**20.69**	7.18	6.72	4.35	5.20	5.18	5.05	**33.68**
13	13th Sept	2.78	4.04	3.65	3.34	4.75	4.78	**23.34**	7.30	6.75	4.40	5.21	5.20	5.07	**33.93**
14	14th Sept	3.08	4.14	3.65	3.50	4.80	3.85	**23.02**	7.54	6.80	4.52	5.37	5.24	5.12	**34.59**
15	15th Sept	3.65	4.14	3.97	4.04	4.94	4.23	**24.97**	8.35	7.30	4.80	5.60	5.42	5.95	**37.42**
16	16th Sept	3.50	4.17	3.92	4.04	4.90	4.18	**24.71**	7.67	6.80	4.54	5.37	5.24	5.12	**34.74**
17	17th Sept	3.10	4.20	3.65	3.65	4.80	3.87	**23.27**	7.78	6.90	4.55	5.37	5.30	5.22	**35.12**
18	18th Sept	3.18	4.20	3.65	3.72	4.80	3.97	**23.52**	7.80	7.00	4.58	5.45	5.30	5.32	**35.45**
19	19th Sept	3.24	4.25	3.65	3.74	4.81	4.00	**23.69**	8.00	7.00	4.63	5.45	5.30	5.4	**35.78**
20	20th Sept	3.27	4.33	3.67	3.78	4.82	4.04	**23.91**	8.37	7.43	4.80	5.60	5.42	6.17	**37.79**
21	21st Sept	3.32	4.45	3.78	3.80	4.82	4.04	**24.21**	8.55	7.43	4.80	5.65	5.42	6.32	**38.17**
22	22nd Sept	2.62	3.45	3.57	2.97	4.60	3.62	**20.83**	7.35	6.75	4.45	5.28	5.20	5.11	**34.14**
23	23rd Sept	2.65	3.49	3.57	2.99	4.60	4.65	**21.95**	7.40	6.75	4.45	5.28	5.20	5.11	**34.19**
24	24th Sept	2.46	3.20	3.37	2.63	4.47	3.40	**19.53**	5.77	6.25	4.60	4.75	4.86	4.77	**31.00**
25	25th Sept	2.47	3.23	3.40	2.67	4.50	3.40	**19.67**	5.85	6.25	4.62	4.79	4.87	4.77	**31.15**
26	26th Sept	2.15	2.82	3.22	2.47	4.37	3.32	**18.35**	5.20	6.03	4.32	4.58	4.80	4.62	**29.55**
27	27th Sept	2.17	2.87	3.24	2.47	4.37	3.32	**18.44**	5.32	6.03	4.34	4.59	4.82	4.67	**29.77**
28	28th Sept	2.18	2.90	3.28	2.50	4.37	3.35	**18.58**	5.35	6.05	4.35	4.62	4.82	4.67	**29.86**
29	29th Sept	2.10	2.50	3.07	2.39	4.25	3.23	**17.54**	4.94	5.70	4.30	4.56	4.75	4.52	**28.77**
30	30th Sept	2.10	2.52	3.10	2.35	4.25	3.23	**17.55**	5.40	6.03	4.40	4.62	4.82	4.67	**29.94**
31	1st Oct	2.09	2.55	3.13	2.35	4.27	3.23	**17.62**	5.00	5.80	4.30	4.56	4.75	4.52	**28.93**
32	2nd Oct	2.20	2.95	3.28	2.50	4.42	3.36	**18.71**	5.47	6.00	4.47	4.62	4.84	4.69	**30.12**
33	3rd Oct	3.37	4.33	3.82	3.88	4.84	4.07	**24.31**	8.05	7.00	4.65	5.47	5.30	5.42	**35.89**

**34**	**4th Oct**	3.55	4.80	3.92	4.00	4.90	4.20	**25.37**	8.10	7.05	4.69	5.54	5.35	8.50	**39.23**
**35**	**5th Oct**	3.43	4.45	3.90	3.99	4.84	4.07	**24.68**	8.12	7.11	4.72	5.54	5.38	5.52	**36.39**
**36**	**6th Oct**	3.92	5.24	4.00	4.11	4.97	4.44	**26.68**	8.72	7.55	4.84	5.75	5.50	6.47	**38.83**
**37**	**7th Oct**	3.97	5.35	3.92	4.14	5.02	4.60	**27.00**	9.03	8,00	5.00	5.90	5.55	6.57	**40.05**
**38**	**8th Oct**	3.99	5.45	3.95	4.15	5.07	4.60	**27.21**	8.96	8.05	5.00	5.95	5.72	6.65	**40.33**
**39**	**9th Oct**	3.99	5.50	3.99	4.15	5.09	4.60	**27.32**	9.06	8.05	5.00	6.00	5.77	6.72	**40.00**
**40**	**10th Oct**	4.45	5.67	4.17	4.30	5.12	4.64	**28.35**	9.15	8.43	6.15	6.72	7.60	7.32	**45.37**
**41**	**11th Oct**	3.95	5.35	4.00	4.13	4.97	4.49	**26.89**	8.84	7.60	4.90	5.76	5.50	6.49	**39.09**
**42**	**12th Oct**	4.03	6.55	4.10	4.15	5.12	4.60	**28.55**	9.07	8.15	5.20	6.50	6.25	6.97	**42.14**
**43**	**13th Oct**	4.00	5.56	4.10	4.16	5.17	4.62	**27.61**	9.07	8.05	5.02	6.00	5.87	6.80	**40.81**
**44**	**14th Oct**	4.05	5.62	4.12	4.18	5.09	4.61	**27.67**	9.08	8.15	5.40	5.83	6.57	7.05	**42.08**
**45**	**15th Oct**	3.43	4.95	3.92	3.99	4.87	4.07	**25.23**	8.17	7.35	4.74	5.55	5.38	5.65	**36.84**
**46**	**16th Oct**	3.90	5.12	3.97	4.06	4.94	4.28	**26.27**	8.92	7.70	4.90	5.83	5.51	6.52	**39.38**
**47**	**17th Oct**	4.05	5.55	4.10	4.18	5.25	4.63	**27.76**	9.12	8.15	5.60	6.60	6.87	7.05	**43.39**
**Mean ±SE of the total Daily milk yield (DMY)**								**22.94 ± 0.47**	**Mean ±SE of the total Daily milk yield (DMY)**						**35.15 ± 0.61**

A1-A6: Buffaloe of group A 1-6; B1-6: Buffaloe of group B

Online resource 3. Physiological assessment: rectal temperature °C (RT) data sheets from 1st Sept to 17th Oct- 2023, study duration for each buffalo of the natural (Gp A) and adaptive group (Gp B)

**Day no.**	**Day/month**	**RT °C/buffalo of Gp A**						**Average RT °C/ gp A**	**RT °C/buffalo of Gp B**						**Average RT °C/ gp A**
		**A1**	**A2**	**A3**	**A4**	**A5**	**A6**		**B1**	**B2**	**B3**	**B4**	**B5**	**B6**	
**1**	**1st Sept**	39.21	39.22	39.12	39.10	39.12	39.10	**39.15**	37.50	37.85	37.35	38.52	38.50	38.00	**37.95**
**2**	**2nd Sept**	39.21	39.12	39.20	39.21	39.22	39.20	**39.19**	37.50	37.60	37.25	38.50	38.50	38.00	**37.89**
**3**	**3rd Sept**	39.12	39.15	39.26	39.22	39.23	39.55	**39.26**	37.55	37.65	37.58	38.54	38.50	38.20	**38.00**
**4**	**4th Sept**	39.31	39.16	39.22	39.23	39.20	39.52	**39.27**	37.53	37.52	37.28	37.53	37.50	37.00	**37.39**
**5**	**5th Sept**	39.22	39.08	39.23	39.24	39.25	39.23	**39.21**	37.50	37.65	37.50	38.20	38.50	38.00	**37.89**
**6**	**6th Sept**	39.21	39.28	39.22	39.22	39.62	39.22	**39.30**	37.50	37.75	37.28	38.20	38.50	38.00	**37.87**
**7**	**7th Sept**	39.23	39.25	39.30	39.25	39.44	39.33	**39.30**	37.50	37.85	37.58	38.20	37.50	38.10	**37.79**
**8**	**8th Sept**	39.25	39.26	39.33	39.32	39.24	39.32	**39.29**	37.50	37.85	37.68	38.10	38.50	38.10	**37.96**
**9**	**9th Sept**	39.26	39.22	39.24	39.20	39.24	39.26	**39.24**	37.50	37.85	37.78	38.20	38.50	38.00	**37.97**
**10**	**10th Sept**	39.13	39.10	39.20	39.52	39.45	39.45	**39.31**	37.50	37.75	37.88	38.10	38.50	38.00	**37.96**
**11**	**11th Sept**	39.17	39.15	39.25	39.22	39.25	39.25	**39.22**	37.50	37.85	37.58	38.00	38.50	38.20	**37.94**
**12**	**12th Sept**	39.18	39.26	39.27	39.20	39.29	39.21	**39.24**	37.50	37.50	37.88	38.20	38.50	38.00	**37.93**
**13**	**13th Sept**	39.12	39.50	39.26	39.25	39.20	39.20	**39.26**	37.50	37.85	37.88	38.20	38.50	38.00	**37.99**
**14**	**14th Sept**	39.11	38.40	38.24	38.23	38.32	38.35	**38.44**	37.50	37.85	37.88	38.20	38.50	38.00	**37.99**
**15**	**15th Sept**	38.52	38.52	38.23	38.22	38.22	38.22	**38.32**	37.50	37.85	37.88	38.20	37.50	38.00	**37.82**

**16**	**16th Sept**	38.18	38.30	38.22	38.25	38.52	38.52	**38.33**	37.50	37.85	37.88	38.20	37.50	38.22	**37.86**
**17**	**17th Sept**	38.13	38.28	38.52	38.52	38.52	38.36	**38.39**	37.50	37.85	37.88	38.20	38.50	38.20	**38.02**
**18**	**18th Sept**	38.52	39.25	38.52	38.52	38.52	38.52	**38.64**	37.50	37.85	37.88	38.20	38.50	38.00	**37.99**
**19**	**19th Sept**	39.22	39.22	39.37	38.52	38.52	38.52	**38.90**	37.50	37.85	37.88	38.20	38.50	38.00	**37.99**
**20**	**20th Sept**	39.13	39.26	39.35	39.21	39.47	39.22	**39.27**	37.50	37.85	37.88	38.20	38.50	38.45	**38.06**
**21**	**21st Sept**	38.52	38.28	38.52	38.52	38.52	38.52	**38.48**	37.50	37.85	37.88	38.20	37.50	38.00	**37.82**
**22**	**22nd Sept**	39.11	39.24	39.28	39.31	39.12	39.22	**39.21**	37.50	37.85	37.88	38.20	38.50	38.00	**37.99**
**23**	**23rd Sept**	39.14	39.22	39.28	39.32	38.52	39.25	**39.12**	37.57	37.85	37.88	38.20	38.50	38.23	**38.04**
**24**	**24th Sept**	39.11	39.22	39.22	39.22	38.52	39.23	**39.09**	37.58	37.85	37.88	38.20	38.50	38.24	**38.04**
**25**	**25th Sept**	39.10	39.22	39.22	39.22	39.23	39.20	**39.20**	37.55	37.85	37.88	38.20	38.50	38.35	**38.06**
**26**	**26th Sept**	39.10	39.22	39.32	39.22	39.32	39.28	**39.24**	37.55	37.85	37.88	38.20	38.50	38.35	**38.06**
**27**	**27th Sept**	39.11	39.22	39.22	39.28	39.33	39.25	**39.24**	37.50	37.85	37.88	38.20	38.50	38.00	**37.99**
**28**	**28th Sept**	39.55	39.22	39.22	39.24	39.24	39.25	**39.29**	37.55	37.85	37.88	38.20	38.50	38.27	**38.04**
**29**	**29th Sept**	39.20	39.22	39.22	39.24	39.25	39.22	**39.23**	37.50	37.85	37.70	38.20	38.50	38.00	**37.96**
**30**	**30th Sept**	39.25	39.24	39.22	39.22	39.24	39.25	**39.24**	37.55	37.35	37.75	38.20	38.50	38.00	**37.89**
**31**	**1st Oct**	39.26	39.28	39.24	39.24	39.25	39.20	**39.25**	37.55	37.75	37.80	38.20	38.50	38.00	**37.97**
**32**	**2nd Oct**	39.25	39.30	39.29	39.29	39.20	39.25	**39.26**	37.55	37.85	37.88	38.20	38.50	38.00	**38.00**
**33**	**3rd Oct**	39.28	39.32	39.30	39.20	39.25	39.27	**39.27**	37.50	37.45	37.68	38.20	38.50	38.00	**37.89**
**34**	**4th Oct**	38.28	38.33	38.26	38.34	38.38	38.35	**38.32**	37.20	37.75	37.58	38.20	38.50	38.00	**37.87**
**35**	**5th Oct**	39.12	39.28	39.27	39.28	39.22	39.20	**39.23**	37.50	37.65	37.68	38.20	38.50	38.25	**37.96**
**36**	**6th Oct**	38.16	39.45	39.34	39.22	39.25	39.28	**39.12**	37.50	37.85	37.38	38.20	37.50	38.10	**37.76**
**37**	**7th Oct**	38.20	39.40	39.32	39.32	39.38	39.35	**39.16**	37.50	37.85	37.28	38.20	37.50	38.00	**37.72**
**38**	**8th Oct**	38.22	39.60	39.12	39.33	39.20	39.25	**39.12**	37.50	37.85	37.28	38.20	37.50	38.10	**37.74**
**39**	**9th Oct**	38.22	38.20	39.21	39.29	39.35	39.25	**38.92**	37.50	37.85	37.48	38.20	37.50	38.10	**37.77**
**40**	**10th Oct**	38.23	38.22	39.22	38.27	38.30	39.25	**38.58**	37.50	37.85	37.35	38.20	38.50	38.00	**37.90**
**41**	**11th Oct**	38.00	38.05	38.02	38.01	38.02	38.03	**38.02**	37.50	37.85	37.45	38.20	38.50	38.20	**37.95**
**42**	**12th Oct**	38.58	38.24	39.26	38.40	38.34	38.32	**38.52**	37.20	37.85	37.45	38.20	38.50	38.00	**37.87**
**43**	**13th Oct**	38.22	38.26	38.29	38.25	38.28	38.26	**38.26**	37.30	37.85	37.88	38.20	38.50	38.00	**37.96**
**44**	**14th Oct**	38.25	38.29	39.33	38.25	38.22	38.23	**38.43**	37.30	37.85	37.25	38.20	38.50	38.00	**37.85**
**45**	**15th Oct**	38.10	38.22	38.25	38.15	38.12	38.25	**38.18**	37.50	37.85	37.88	38.20	38.50	38.00	**37.99**
**46**	**16th Oct**	38.20	38.55	38.12	38.10	38.25	38.57	**38.30**	37.65	37.80	37.55	38.00	38.00	38.20	**37.87**
**47**	**17th Oct**	38.10	38.23	38.22	38.15	38.15	38.22	**38.18**	37.55	37.82	37.56	38.10	37.85	38.10	**37.83**
**Mean ±SE of Rectal Temperature (RT) °C/ gp A**								**38.93± 0.01**							**37.91± 0.04**

RT: Average Rectal Temperature (° C)

Online resource 4. Physiological assessment: respiratory rate (RR) (Beat Per Minute, BPM) from 1st Sept to 17th Oct- 2023, study duration for each buffalo of the natural (Gp A) and adaptive group (Gp B)

Day no.	Day/month	RR (bpm) buffalo of Gp B						Average RR/ gp A (bpm)	RR (BPM) buffalo of Gp B						Average RR/ gp B (bpm)
		A1	A2	A3	A4	A5	A6		B1	B2	B3	B4	B5	B6	
1	1st Sept	29	29	28	28	29	30	**28.83**	24	24	25	25	25	25	**24.67**
2	2nd Sept	28	27	28	28	27	28	**27.67**	23	23	24	24	25	25	**24.00**
3	3rd Sept	27	27	29	28	30	29	**28.33**	24	24	23	25	25	25	**24.33**
4	4th Sept	28	30	30	27	28	28	**28.50**	24	24	24	25	25	25	**24.50**
5	5th Sept	29	28	29	29	29	29	**28.83**	23	24	24	25	25	25	**24.33**
6	6th Sept	27	29	28	30	28	29	**28.50**	22	23	24	24	25	25	**23.83**
7	7th Sept	29	29	29	27	28	29	**28.50**	22	24	24	25	25	24	**24.00**
8	8th Sept	29	28	28	30	29	30	**29.00**	23	23	24	25	25	24	**24.00**
9	9th Sept	29	30	28	30	28	30	**29.17**	22	23	23	24	24	24	**23.33**
10	10th Sept	28	29	28	28	28	30	**28.50**	23	22	24	23	25	24	**23.50**
11	11th Sept	29	29	28	28	29	28	**28.50**	24	23	24	24	24	24	**23.83**
12	12th Sept	29	28	29	28	29	28	**28.50**	23	23	24	24	24	24	**23.67**
13	13th Sept	29	29	28	29	28	29	**28.67**	22	24	23	25	24	24	**23.67**
14	14th Sept	27	26	26	27	26	27	**26.50**	23	23	24	23	24	24	**23.50**
15	15th Sept	26	26	26	26	26	27	**26.17**	22	23	23	24	24	24	**23.33**
16	16th Sept	27	26	26	26	26	27	**26.33**	22	22	23	23	23	24	**22.83**
17	17th Sept	25	25	26	25	27	27	**25.83**	22	23	22	24	23	23	**22.83**
18	18th Sept	28	29	29	28	30	30	**29.00**	24	24	24	24	24	23	**23.83**
19	19th Sept	29	30	30	27	30	29	**29.17**	24	24	24	24	24	23	**23.83**
20	20th Sept	28	29	27	27	30	28	**28.17**	24	24	23	23	24	24	**23.67**
21	21st Sept	27	28	27	26	27	28	**27.17**	23	24	23	22	24	25	**23.50**
22	22nd Sept	29	29	29	28	30	30	**29.17**	24	23	24	23	23	24	**23.50**
23	23rd Sept	29	29	28	29	29	29	**28.83**	23	23	24	24	24	23	**23.50**
24	24th Sept	28	29	29	29	29	29	**28.83**	23	22	24	25	24	24	**23.67**
25	25th Sept	28	29	28	28	29	29	**28.50**	22	23	24	24	24	24	**23.50**
26	26th Sept	29	30	29	29	30	29	**29.33**	21	24	23	24	25	23	**23.33**
27	27th Sept	29	29	29	29	30	29	**29.17**	23	24	24	24	23	23	**23.50**
28	28th Sept	29	29	29	29	29	28	**28.83**	24	25	23	24	24	25	**24.17**
29	29th Sept	30	30	29	29	30	30	**29.67**	24	25	22	24	24	25	**24.00**
30	30th Sept	29	30	30	29	30	29	**29.50**	24	24	24	22	24	25	**23.83**
31	1st Oct	29	29	29	30	29	30	**29.33**	22	24	23	23	24	25	**23.50**
32	2nd Oct	29	29	29	28	29	29	**28.83**	22	23	24	25	24	23	**23.50**

**33**	**3rd Oct**	29	29	29	28	29	29	**28.83**	23	24	24	24	25	24	**24.00**
**34**	**4th Oct**	27	27	27	27	27	27	**27.00**	24	25	24	24	24	25	**24.33**
**35**	**5th Oct**	27	28	28	27	27	27	**27.33**	24	25	24	24	25	24	**24.33**
**36**	**6th Oct**	27	27	27	27	27	28	**27.17**	22	24	23	24	23	23	**23.17**
**37**	**7th Oct**	27	27	27	26	26	26	**26.50**	21	24	23	23	22	22	**22.50**
**38**	**8th Oct**	27	27	26	27	27	27	**26.83**	23	24	22	23	23	23	**23.00**
**39**	**9th Oct**	28	27	27	27	27	26	**27.00**	22	23	24	23	23	23	**23.00**
**40**	**10th Oct**	27	27	27	27	26	25	**26.50**	24	24	23	25	25	24	**24.17**
**41**	**11th Oct**	25	26	26	26	26	27	**26.00**	24	25	25	24	25	25	**24.67**
**42**	**12th Oct**	27	27	27	26	27	27	**26.83**	23	23	24	24	24	25	**23.83**
**43**	**13th Oct**	28	27	26	27	27	27	**27.00**	22	23	23	23	24	25	**23.33**
**44**	**14th Oct**	27	27	27	27	26	26	**26.67**	23	25	23	24	24	24	**23.83**
**45**	**15th Oct**	27	27	28	27	27	27	**27.17**	24	23	25	24	24	25	**24.17**
**46**	**16th Oct**	27	27	27	27	27	26	**26.83**	25	24	24	23	24	24	**24.00**
**47**	**17th Oct**	27	27	26	26	26	27	**26.50**	24	24	24	24	24	25	**24.17**
**Mean ±SE of RR (bpm)/ gp A**								**27.96± 0.16**	**Mean ±SE of RR (bpm)/ gp B**						**23.73 ± 0.07**

RR (bpm): Respiratory rate (beat per minute)
